# Targeting mitochondrial DNA polymerase gamma for selective inhibition of MLH1 deficient colon cancer growth

**DOI:** 10.1371/journal.pone.0268391

**Published:** 2022-06-03

**Authors:** Berna Somuncu, Aysegul Ekmekcioglu, Fatma Merve Antmen, Tugce Ertuzun, Emre Deniz, Nazli Keskin, Joon Park, Ilgu Ece Yazici, Busra Simsek, Batu Erman, Whitney Yin, Burak Erman, Meltem Muftuoglu

**Affiliations:** 1 Department of Medical Biotechnology, Institute of Health Sciences, Acibadem Mehmet Ali Aydinlar University, Istanbul, Turkey; 2 Department of Molecular Biology and Genetics, Faculty of Arts and Sciences, Acibadem Mehmet Ali Aydinlar University, Istanbul, Turkey; 3 Molecular Biology, Genetics and Bioengineering Program, Faculty of Engineering and Natural Sciences, Sabanci University, Istanbul, Turkey; 4 Department of Pharmacology and Toxicology, University of Texas Medical Branch, Galveston, Texas, United States of America; 5 Department of Molecular Biology and Genetics, Faculty of Arts and Sciences, Bogazici University, Istanbul, Turkey; 6 Department of Chemical and Biological Engineering, Koc University, Istanbul, Turkey; University of South Alabama Mitchell Cancer Institute, UNITED STATES

## Abstract

Synthetic lethality in DNA repair pathways is an important strategy for the selective treatment of cancer cells without harming healthy cells and developing cancer-specific drugs. The synthetic lethal interaction between the mismatch repair (MMR) protein, MutL homolog 1 (MLH1), and the mitochondrial base excision repair protein, DNA polymerase γ (Pol γ) was used in this study for the selective treatment of MLH1 deficient cancers. Germline mutations in the *MLH1* gene and aberrant *MLH1* promoter methylation result in an increased risk of developing many cancers, including nonpolyposis colorectal and endometrial cancers. Because the inhibition of Pol γ in MLH1 deficient cancer cells provides the synthetic lethal selectivity, we conducted a comprehensive small molecule screening from various databases and chemical drug library molecules for novel Pol γ inhibitors that selectively kill MLH1 deficient cancer cells. We characterized these Pol γ inhibitor molecules *in vitro* and *in vivo*, and identified 3,3’-[(1,1’-Biphenyl)-4’,4’-diyl)bis(azo)]bis[4-amino-1-naphthalenesulfonic acid] (congo red; CR; Zinc 03830554) as a high-affinity binder to the Pol γ protein and potent inhibitor of the Pol γ strand displacement and one-nucleotide incorporation DNA synthesis activities *in vitro* and *in vivo*. CR reduced the cell proliferation of MLH1 deficient HCT116 human colon cancer cells and suppressed HCT116 xenograft tumor growth whereas it did not affect the MLH1 proficient cell proliferation and xenograft tumor growth. CR caused mitochondrial dysfunction and cell death by inhibiting Pol γ activity and oxidative mtDNA damage repair, increasing the production of reactive oxygen species and oxidative mtDNA damage in MLH1 deficient cells. This study suggests that the Pol γ inhibitor, CR may be further evaluated for the MLH1 deficient cancers’ therapy.

## Introduction

The cytotoxic effects of most chemotherapeutic agents and radiation therapy are related to their ability to induce DNA damage. The DNA-repairing systems of cancer cells counteract these forms of treatment and are good candidates for novel targets for cancer therapy. Conversely, DNA repair deficiency caused by loss-of-function mutations in DNA repair genes that have also tumor suppressor gene function leads to cancer. Cancer cells thus become dependent on backup/complementary DNA repair pathways for survival and proliferation. DNA repair-based synthetic lethal targeted therapy depends on inhibiting these backup/complementary DNA repair pathways in repair-deficient cancer cells treated with specific DNA damaging chemotherapeutic agents. This kind of therapy could ideally selectively kill tumor cells while sparing normal cells and reduce potential off-target side effects, for example, the clinically-used poly(ADP-ribose) polymerase 1 (PARP1) inhibitors in the selective treatment of breast cancer 1/2 (BRCA1/2) deficient cancers [1, 2].

The synthetic lethal interaction between the mismatch repair (MMR) protein, MLH1, and the mitochondrial base excision repair (BER), mitochondrial MMR and mtDNA replication protein, DNA polymerase γ (Pol γ) [3–5] was used in the current study for the selective treatment of MLH1 deficient cancers. Because MMR deficient cancers are resistant to a wide range of standard chemotherapeutic agents including platinum compounds and methylating agents, there is an urgent need to develop new drugs and strategies for the treatment of these cancers. Germline mutations in the *MLH1* gene and aberrant *MLH1* promoter methylation result in a deficiency of the MMR pathway and an increased risk of developing numerous cancers, including nonpolyposis colorectal cancer (Lynch syndrome), endometrial, and ovarian cancers [6].

MLH1 is a tumor suppressor protein where tumor cells can have a complete loss of MLH1 function but normal cells mostly retain at least one functional *MLH1* allele and thereby having a functional MMR pathway [7–9]. Moreover, the MMR pathway also plays a role in the repair of oxidative DNA damage besides its major role in repairing base mispairs arising during DNA replication [4, 10–13]. It has been suggested that the MMR proteins involved in the mitochondrial MMR could be distinct from that of nuclear MMR [4]. de Souza-Pinto et al. showed that the repair factor Y-box binding protein (YB-1) is localized to mitochondria and required for the mitochondrial MMR activity [4]. It has also been shown that the mitochondrial extracts of MSH2 deficient cells have mismatch binding activity, suggesting that the key nuclear MMR protein, MSH2, does not contribute to the mismatch binding activity in mitochondria [4, 14]. Another key MMR protein, MLH1 is shown to be localized to the mitochondria and required for the mitochondrial MMR pathway [3, 15]. All the mitochondrial MMR factors have not been identified yet. The Ashworth group demonstrated that silencing of the *Pol γ* gene in MLH1 deficient cancer cells resulted in selective death of the MLH1 deficient cancer cells, which was associated with the accumulation of oxidative mtDNA lesions. The same group also showed that MSH2 deficiency is synthetically lethal with inhibition of DNA polymerase β (Pol β), but not Pol γ. Thus, it has been suggested that small molecules that inhibit the activity of Pol γ protein may be an effective therapeutic approach for the selective treatment of MLH1 deficient tumors [3].

BER is the major pathway for the repair of oxidative DNA lesions and is present in both nucleus and mitochondria by similar mechanisms that share many of the core BER enzymes. Briefly, both nuclear and mitochondrial BER pathways include four distinct steps; the pathway is initiated by recognition and removal of damaged base, followed by end processing and then gap filling with a DNA polymerase and end with a nick sealing by a DNA ligase. The replicative mtDNA polymerase, Pol γ, involves in the synthesis step of mitochondrial BER, and Pol β involves in the synthesis step of nuclear BER [5]. Recent studies have shown that nuclear BER protein, Pol β, is also located in mitochondria and could play a role in mitochondrial BER and mtDNA integrity [16–18]. The mechanism of Pol β function in mitochondrial genome maintenance is under further investigation. Pol γ is a heterotrimeric mtDNA polymerase enzyme that consists of a homodimer of accessory subunits (Pol γB) and a catalytic subunit (Pol γA). The catalytic subunit Pol γA has polymerase, deoxyribose phosphate lyase, and 3′-5′ exonuclease activities. Accessory subunits, Pol γB have no intrinsic enzymatic activity but instead increase the processivity of the Pol γA subunit [19]. Nucleoside reverse transcriptase inhibitors (NRTIs) are well-known and extensively studied inhibitors of Polγ [20]. The cytosine-based nucleoside analogs such as cytarabine and menadione (vitamin K_3_) known to inhibit Pol γ are selectively cytotoxic to MLH1 and MSH2 deficient cancer cells [21–24]. The known Pol γ inhibitors have not reached the clinical stage to target MLH1 deficient cancers. We have therefore conducted a comprehensive small molecule screen from various databases and chemical drug library molecules for novel inhibitors of the Pol γ protein that selectively kill MLH1 deficient cancer cells. We characterized these Pol γ inhibitor molecules *in vitro* and *in vivo*, and present 3,3’-[(1,1’-Biphenyl)-4’,4’-diyl)bis(azo)]bis[4-amino-1-naphthalenesulfonic acid] (congo red; CR; Zinc 03830554) that is a sulfonated diazo dye as a high affinity binder and inhibitor of the Pol γ enzyme with specific cytotoxicity against MLH1 deficient colon cancer cells and tumors. The findings in this paper are of both biochemical and clinical interest.

## Materials and methods

### Molecular docking and surface plasmon resonance analysis

Three-dimensional coordinates of the X-ray crystal structure of the human Pol γ holoenzyme (protein data bank identification code (PDB ID): 3IKM) were selected as the receptor models in docking programs. The binding models for Pol γ inhibitors were predicted by using GOLD (GOLD Suite v5.2.2) on a Windows server equipped with an Intel Core i7-4600U processor (2.7 GHz) and 8 GB of RAM. The library used in molecular docking was composed of about 8550 plant secondary metabolites that provide 3D structures that we obtained from various online available databases: KEGG phytochemical compounds (https://www.genome.jp/kegg-bin/get_htext?br08003.keg), Analyticon-discovery MEGxp pure natural plant products (https://ac-discovery.com/purified-natural-product-screening-compounds), seaweed metabolites database (SWMD) (https://www.swmd.co.in), indofine chemicals (https://indofinechemical.com), Indian plant anticancer compounds database (InPACdb) (http://www.inpacdb.org), zinc database (https://zinc.docking.org), zinc natural products (https://zinc15.docking.org/substances/subsets/natural-products), and zinc database FDA approved drugs (http://zinc.docking.org/substances/subsets/fda). Docking of molecules was performed using the GOLD software and the hit molecules were evaluated according to the binding free energies (-10 ≤ kcal/mol).

SPR studies were performed as a service by Prof. Aykut Uren’s group at Georgetown University, USA [25]. Human recombinant Pol γ protein (Chimarx, USA) was immobilized on Biacore T100 CM5 sensor chips (GE Healthcare, Piscataway, NJ, USA), and 1200 small molecules from the Prestwick Chemical Library (http://www.prestwickchemical.com) were injected at a single concentration for initial screening. The relative binding of each compound (response units, RU) to Pol γ and RNA helicase A were normalized to Rmax, which was taken as 100%. In addition, 7 different concentrations of the small molecules from *in silico* screening with calculated binding free energies around -10 kcal/mol were injected over the surface in triplicates. The small molecules were synthesized as a service and purchased from Ambinter (www.ambinter.com). SPR sensorgrams and dissociation constants (K_D_) values were obtained using Biacore T100 software.

### Pol γ activity assays

Pol γA catalytic subunit (140 kDa) and Pol γB accessory subunit (55 kDa) were expressed and purified from insect Sf9 cells and *E*. *coli*, respectively, as described previously [19]. Pol γ holoenzyme (exonuclease deficient) was formed by the combination of Pol γA and Pol γB monomers at a 1:2 molar ratio. Pol γ activities were measured using a 51mer duplex oligodeoxynucleotide containing 1-nt gap substrate at position 26. All oligonucleotides were purchased from DNA Technology, Denmark, and the sequences are; G1: 5′-GCTTAGCTTGGAATCGTATCATGTA-3′, G2: 5′-ACTCGTGTGCCGTGTAGACCGTGCC-3′, G3: 5′-GGCACGGTCTACACGGCACACGAGT**G**TACATGATACGATTCCAAGCTAAGC-3′. G1 substrate was 5′-end-labeled using [γ-^32^P]ATP (Perkin Elmer Life Sciences) and T4 polynucleotide kinase (New England Biolabs) as described before [26]. The activity reactions were done as previously described [27] with some modifications. Pol γ (40 nM) was incubated with the compounds as indicated in the figure legends on ice for 5 min in a reaction buffer (20 mM HEPES/KOH pH.7.5, 0.1 mg/ml BSA, 100 mM KCl, 1 mM β-mercaptoethanol, and 5% glycerol). Strand displacement DNA synthesis reactions (10 μl) were initiated by the addition of 100 fmol ^32^P-labeled 1 nt-gap substrate, 5 mM MgCl_2_ and 0.05 mM dNTPs (dATP, dCTP, dGTP and dTTP) to Pol γ and the compound reaction mixture and incubated at 37°C for 20 min. The same reaction conditions were used for the 1-nt gap-filling assay, except dCTP was used instead of dNTPs. Reactions were stopped by the addition of an equal amount of the formamide loading dye (90% formamide, 10 mM EDTA, 0.01% bromphenol blue, and 0.01% xylene cyanol). The samples were incubated at 95°C for 10 min and then run on 20% polyacrylamide-7 M urea gel. Gels were visualized by PhosphorImager (Typhoon FLA9500) and analyzed using the ImageQuant software (GE Healthcare Life Sciences). The experiments were done in triplicate. The percent of each product with a given number of nucleotides incorporated was calculated as follows: % of total = (amount of radioactivity associated with each dNMP addition/total radioactivity) x 100.

### Cell lines and culture

HCT116VA (hMLH1 mutant; vector alone) and HCT116V1 (hMLH1 transfected) cells were kind gifts from Dr. Anatoly Zhitkovich at Brown University, USA. MMR deficient colon cancer cell lines- HCT116 (MLH1 deficient), Lovo (hMSH2 mutant), and MCF7 breast cancer cell lines were obtained from the American Type Culture Collection (ATCC). HCT116 and MCF7 cell lines were grown in DMEM (Gibco-Life Technologies) and Lovo cell lines were grown in RPMI (Gibco-Life Technologies) supplemented with 10% fetal bovine serum (Gibco-Life Technologies) and 1% Pen/Strep (Gibco-Life Technologies). The medium for HCT116VA and HCT116V1 additionally contained 400 μg/ml Geneticin (G418, Life Technologies Invitrogen).

### Isolation of mitochondria from HCT116 cells and cell extract single nucleotide gap-filling assay

HCT116 cells (VA and V1) were incubated with 50 μM CR in serum-free Dulbecco’s modified eagle medium for 2 h at 37°C, 5% CO_2_ incubator. Isolation of mitochondria from treated and untreated HCT116 cells was performed as previously described [28]. The cell extracts using both the mitochondrial and nuclear pellets were prepared as previously described [28]. Protein concentration was determined by the Bradford method using bovine serum albumin (BSA) as a standard (Bio-Rad Protein Assay, Bio-Rad Laboratories). The purity of the mitochondrial and nuclear extracts was confirmed by western blotting using the following primary antibodies: mouse anti-Lamin A/C (1:200, cat # NCL-LAM-A/C, Leica Biosystems) and mouse anti-COX IV (1:1000, cat # A21347, Invitrogen). After incubating with anti-mouse IgG, HRP linked (1:5000, cat # 7076, Cell Signaling Technology) secondary antibody, the membrane was exposed to Pierce ECL Plus according to the manufacturer’s protocol (Pierce). The immunoblots were then visualized using ChemiDoc MP Imaging systems (Bio-Rad Laboratories).

Single nucleotide gap-filling reactions using HCT116 mitochondrial and nuclear cell extracts were performed as described before [26] with some modifications. Single nucleotide gap-filling reactions were performed in a 10 μl reaction buffer containing 100 fmol 1-nt gap substrate and 2 μCi of ^32^P-dCTP (GE Healthcare, USA). Reactions were initiated by adding 0.5 μg of mitochondrial and nuclear extracts and were incubated at 37°C for 1h. After the addition of the equal volume of the formamide stop dye, reactions were incubated at 75°C for 10 min. Samples were run on 20% PAGE-Urea gel and visualized as described above. The experiments were performed in duplicate. The incorporation of ^32^P-dCTP activity was quantified as the increase in the signal intensity.

### Generation of Pol γ knockout HCT116 cells lines by CRISPR/Cas9 and Pol γ shRNA

A guide RNA (gRNA) targeting a HpyCH4III restriction enzyme cut site located downstream of the translation start site of the human *POLGA* gene (ENSG00000140521) was selected by using CRISPOR webtool [29]. Twenty nucleotides long gRNA was initially synthesized as complementary oligonucleotide pairs with flanking sequences for DNA cloning (CRISPR#6 top strand: cac cgA ACC GGC CCT GGC CCG ACG G and bottom strand: aaa cCC GTC GGG CCA GGG CCG GTT c). Complementary oligonucleotides were annealed, phosphorylated, and then cloned into the pSpCas9(BB)-2A-Puro plasmid (PX459 was a gift from Dr. Feng Zhang (Addgene plasmid # 48139)) digested with BbsI [30]. Plasmids were transfected into HCT116 cells by using a polyethyleneimine protocol (cat # 23966, Polysciences) as previously described [31]. Twenty-four hours post-transfection, growth media was supplemented with 1 mM of each sodium pyruvate (cat # 11360, Gibco) and uridine (cat # U3003, Sigma), and 48 h post-transfection, transfected cells were briefly selected with puromycin (cat # P9620, Sigma) for another 48 h. Single-cell clonal expansions were performed and emerging cell clones were first assayed by restriction fragment length polymorphism using the HpyCH4III restriction enzyme, which recognizes a site overlapping the CRISPR/Cas9 target locus. The target genomic locus was PCR-amplified (Polγ forward: TTG GGG ACG CAG TAA ATG C and Polγ reverse: AGT GCT GGT CCA GGT TGT C) and digested with HpyCH4III. Clones with undigested PCR products were additionally analyzed by Western blotting against the POLGA protein using an anti-POLGA primary antibody (1:1000, cat # 97661, Abcam) and an HRP linked anti-rabbit IgG, secondary antibody (1:5000, cat # 7074, Cell Signaling Technology). Loading control, β-Actin (8H10D10) Mouse mAb (1:1000, cat # 3500, Cell Signaling Technology), and secondary antibody HRP linked anti-mouse IgG (1:5000, cat # 7076, Cell Signaling Technology) was used. Single-cell clones negative for POLGA protein expression were further characterized by Sanger DNA sequence analysis of PCR products spanning the edited site, which were TA-cloned (cat # K1214, Thermo Scientific). To identify all possible mutant alleles, -16 TA-plasmid clones per cell clone were Sanger sequenced using Applied Biosystems 3500 Genetic Analyzers according to the manufacturer’s protocol.

HCT116V1 cells were transfected with 50 nM shRNA targeting Pol γ catalytic subunit (OriGene, cat no.TR310307) and control shRNA (OriGene, cat no. TR30012) using FuGENE HD transfection reagent (Promega) according to the manufacturer’s instructions. Knockdown levels of Pol γ protein were detected by Western blot analysis as described above.

### Cytotoxicity assay using the xCELLigence DP system

Impedance-based real-time detection of cell proliferation and cytotoxicity experiments were performed according to the instruction manual of the xCELLigence DP system (Acea Biosciences). The presence of MLH1 protein in HCT116VA and HCT116V1 cells was tested by Western blot analysis. The whole-cell extracts were prepared using radioimmunoprecipitation buffer and Western blot analysis were performed as previously described [26]. The immunoblots were analyzed with MLH1 (4C9C7) Mouse mAb (1:1000, cat # 3515, Cell Signaling Technology) and β-Actin (8H10D10) Mouse mAb (1:1000, cat # 3500, Cell Signaling Technology) primary antibodies and anti-mouse IgG, HRP linked (1:5000, cat # 7076, Cell Signaling Technology) secondary antibody, and then the membrane was exposed to Pierce ECL Plus according to the manufacturer’s protocol (Pierce). The immunoblots were then visualized using ChemiDoc MP Imaging systems (Bio-Rad Laboratories). After determining the optimum HCT116 cell number from its proliferation pattern, 15000 HCT116VA, HCT116V1, CRISPR/Cas9 HCT116 Pol γ knockout, and HCT116 control (CTRL; vector control) cells/well; 30000 Lovo cells/well, and 5000 MCF7 cells/well were seeded in an E-Plate 16. Approximately 22 h after seeding, when the cells were in the log growth phase the cells were treated with different concentrations of compounds and monitored every 30 min for 70–72 h. The cells were treated with a final concentration of 0.02% DMSO which served as vehicle control. The results were expressed by a normalized cell index (CI), which is derived from the ratio of CIs before and after the addition of compounds. Normalized CI was plotted as the mean value from duplicates; error bars represent the SD of the mean. The Real-Time Cell Analysis (RTCA) software was used to calculate IC_50_ values from the dose-response curve at 48 h (post-CR administration).

### Clonogenic assay

HCT116VA and HCT116V1 cells were seeded in 6-well plates (500 cells/well) and treated with 5 μM CR. Colonies were allowed to grow for 10 days. Then, the plates were washed with 1XPBS, fixed with methanol, stained with 1% methylene blue, and washed with 1XPBS to remove excess dye, air dried, and counted. Percent survival fraction was expressed as the ratio of plating efficiency (number of colonies formed/number of colonies were seeded) of treated cells to that of untreated control cells multiplied by 100.

### Reactive Oxygen Species (ROS) measurements

A cellular ROS assay kit (Abcam) was used to measure cellular ROS according to the manufacturer’s instructions. Briefly, HCT116VA and HCT116V1 cells (5x10^3^ cells per well) were plated in clear bottom black-walled 96-well plates. After 24 h the cells were treated with 1 and 5 μM CR alone or in combination with the ROS scavenger 1 mg/ml N-Acetyl cysteine (NAC; Sigma) for 48 h. Then the cells were incubated for 20 μM H2DCFDA for 45 min at 37°C CO_2_ incubator. ROS levels were measured at Ex/Em 485/535 nm using Varioskan Multimode Microplate Reader (ThermoFisher Scientific). The experiment was repeated three times. Fluorescence data were normalized to the corresponding cell viability data, which was measured using CellTiter-Glo Luminescent Cell Viability assay (Promega) according to the manufacturer’s instructions.

ROS levels were also measured using flow cytometry. A total of 6x10^3^ HCT116VA and HCT116V1 cells were seeded in 6 well plates for 24 h. The culture medium (described above) was exchanged with DMEM (FBS, Pen/Strep/Phenol red free) and incubated with H2DCFDA at a final concentration of 10 μM for 40 min. CR at 0, 1, and 5 μM was added after removal of H2DCFDA containing media and incubated for 1 h. Single cell suspensions (0.25% Trypsin) were analyzed for H2DCFDA fluorescence on a BD Accuri C6 instrument (BD Biosciences) using 488nm excitation. Fluorescence from the Fl1 channel was analyzed using FlowJo 10.8 software.

### Quantitative analysis of 8-hydroxydeoxyguanosine (8-OHdG)

Mitochondria from treated and untreated HCT116VA and HCT116V1 cells were isolated as described above. MtDNA from isolated mitochondria was purified using a DNeasy kit (Qiagen). 8-OHdG was determined by using Oxiselect Oxidative DNA Damage ELISA Kit (8-OHdG Quantitation; CellBioLabs) according to the manufacturer’s instructions. Results were calculated according to the standard curve. The detection range was 100 pg/ml-20 ng/ml.

### mtDNA copy number

MtDNA copy number was determined by amplifying the mitochondrial MT-ND1 gene for mtDNA relative to the GAPDH gene for nDNA. Total DNA was isolated from HCT116VA and HCT116V1 cells treated with 20 μM CR or 0.01% DMSO for 8 h and 24 h using QIAamp DNA mini kit (Qiagen) according to the manufacturer’s instructions. Real-time quantitative PCR (qPCR) was performed using Fast start essential DNA Green master mix (Roche Diagnostics, USA) according to the manufacturer’s instructions with annealing temperature 60 using LightCycler Nano instrument (Roche Diagnostics, USA). The primer sequences are: MT-ND5: 5′- CCGGAAGCCTATTCGCAGGA -3′; 5′- ACAGCGAGGGCTGTGAGTTT -3′; GAPDH: 5′-GTGCTCCCACTCCTGATTTCT-3′; 5′-ACCCCTTCTCTAAGTCCCTCCT-3′. MtDNA copy number was calculated by 2^-ΔCt^, where ΔCt = nDNA Ct–mtDNA Ct. The fold change in mtDNA copy number due to treatment was calculated by 2^-ΔΔCt^ method [32]. Relative mtDNA content = 2x2^ΔCt^ [33]. Each experiment was performed in triplicate.

### Xenograft tumors

A total of 5x10^6^ HCT116, HCT116V1 or Lovo cells were suspended in 2:1 mixture of PBS:Matrigel (BD Biosciences), and 100 μl of the mixture was injected subcutaneously into the lower back region of 6-week-old female athymic nude mice (Crl:NU(NCr)-Foxn1; Charles River Laboratories, Germany). The tumor size was measured with calipers. When the tumors have reached a volume of 90–110 mm^3^, the mice were injected intraperitoneally with compound or vehicle (DMSO 0.02%) alone. They were administrated intraperitoneally every day until the tumor volume is around 1200 mm^3^ (approximately 3 weeks). Tumor volumes were calculated according to the formula: volume = (length x width^2^)/2. To monitor compound toxicity levels, the body weights of the mice were measured every day. After 3 weeks, the mice were euthanized by the ketamine-xylazine anesthetic combination followed by cervical dislocation. To prevent/alleviate animal suffering, animals were euthanized in a timely manner by professional veterinarians [34]. This study was approved by Acibadem Mehmet Ali Aydinlar University’s local ethics committee for animal experiments (ACU-HADYEK 2015/30).

### Statistical analysis

All quantitative data are presented as the means ± standard deviation (STD) of at least three replicates. Statistical analysis was performed using a two-tailed Student’s t-test for pairwise comparison and one-way ANOVA with multiple comparisons by Tukey post-hoc tests for multiple comparisons. Statistical analysis was performed using the GraphPad Prism 9.2.0 software. p < 0.05 was considered statistically significant.

## Results

### Screening of small molecules for direct binding to Pol γ and to inhibit its activity

To identify a potent Pol γ small molecule inhibitor, we screened the Prestwick Chemical Library molecules by Surface plasmon resonance (SPR) technology for direct binding to Pol γ holoenzyme (S1A Fig). The Prestwick Chemical Library molecules include 1200 off-patent small molecules, 99% of which are approved by the United States Food and Drug Administration, the European Medicines Agency, and other agencies. The analyte binding capacity or theoretical maximum response (Rmax) of each compound was calculated for both Pol γ and RNA helicase A, which was used as a negative control protein to eliminate nonspecific binders and false-positive results. Molecules that bound Pol γ more than an arbitrary threshold of 3-fold compared to the negative control protein were designated as primary hits (S1A Fig, red dots in sensorgrams). As a result, 13 primary hits were identified out of 1200 small molecules that demonstrated various degrees of specific binding to Pol γ (S1A Fig, red dots in sensorgrams, and S1 Table).

**Fig 1:**
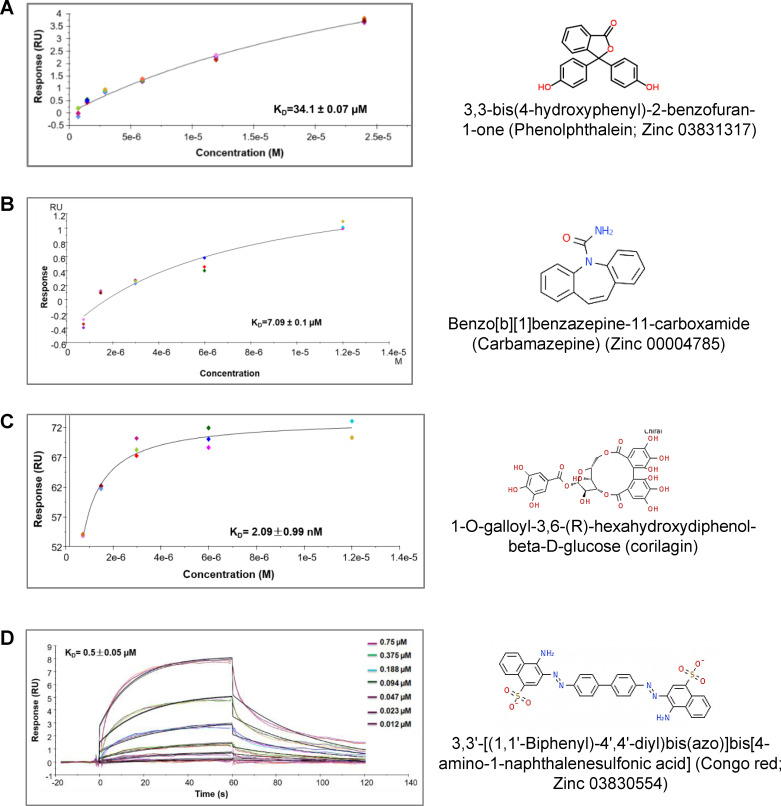
Screening of small molecules for direct binding to Pol γ by SPR technology. (A-D) SPR binding kinetics of computationally predicted small molecules to Pol γ. Sensorgrams show the interaction between compounds and Pol γ at different concentrations and K_D_ values. All data are presented as the means ± standard deviation (STD) of three independent experiments. The chemical structures of small molecules are given.

To increase the pool of primary hits, we also used an *in silico* structural based molecular docking approach, using the available crystal structure of Pol γ holoenzyme (PDB ID: 3IKM) and screened 8550 plant secondary metabolites from an in house *in-silico* library of drug-like compounds. We targeted the catalytic subunit of Pol γ, Pol γA. SPR kinetic analysis revealed that four computationally predicted small molecules bound directly to Pol γ with high affinity. The equilibrium dissociation constant (K_D_ or affinity) of these molecules is ranging from higher 34.1±0.07 μM to 2.09±0.99 nM (Fig 1A–1D). Three of these compounds (Fig 1A, 1B and 1D) are from the Zinc database: 3,3’-[(1,1’-Biphenyl)-4’,4’-diyl)bis(azo)]bis[4-amino-1-naphthalenesulfonic acid] (congo red; CR; Zinc 03830554), benzo[b][1]benzazepine-11-carboxamide (carbamazepine; Zinc 00004785), and 3,3-bis(4-hydroxyphenyl)-2-benzofuran-1-one (phenolphthalein; Zinc 03831317), and one compound, corilagin (C10219) is from the Kyoto Encyclopedia of Genes and Genomes (KEGG) drug library (Fig 1C). The association rate constant (ka or k_ON_ = 5.25E+4 1/Ms), dissociation rate constant (kd or koff = 0.029 1/s), K_D_ (0.5 ± 0.05 μM) for CR were calculated using Biacore T100 software. The thymidine analog fialuridine (a known binder to Pol γ) [35] was used as a positive control and bound to Pol γ with an affinity of K_D_ 6.83 ± 2.3 μM (S1B Fig).

**Fig 2:**
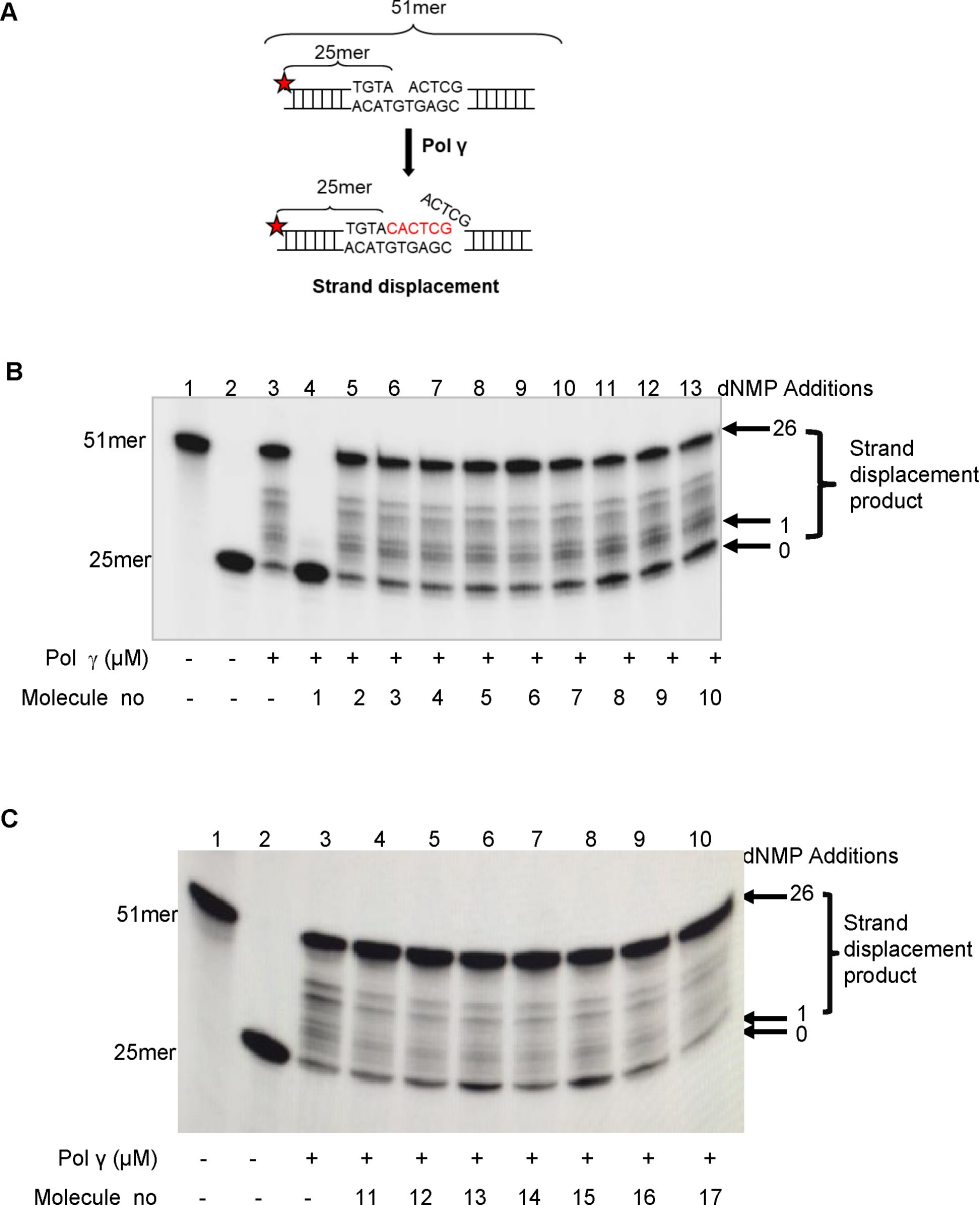
Screening the small molecules on the strand displacement DNA synthesis activity of Pol γ. (A) Schematic of the ^32^P-labeled (red star) 51 bp DNA substrate containing 1-nt gap at position 26 is shown before and after Pol γ strand displacement activity. Reactions contained the substrate and Pol γ alone (40 nM; lane 3) or together with 20 μM of the small molecules. (B) Molecule no 1–10: CR, corilagin, carbamazepine, phenolphthalein, acemetacin, indomethacin, amikacin hydrate, chenodiol, econazole nitrate, and ketoconazole, respectively. (C) Molecule no 11–17: Nicardipine hydrochloride, miconazole, nialamide, nystatin, misoprostol, pimethixene maleate, and rifampicin, respectively. Lane 1, 51mer oligodeoxynucleotide; lane 2, substrate alone.

In order to identify molecules that inhibit the biochemical activity of the Pol γ enzyme, we performed *in vitro* activity assays with purified Pol γ protein. We first screened the 17 Pol γ binder small molecules (Fig 1A–1D and S1A Fig) on their effects on the strand displacement DNA synthesis activity of Pol γ using a 51mer DNA substrate, containing a 1-nt gap at position 26 (Fig 2). Four molecules from the *in-silico* library with the highest Pol γ binding affinity (Fig 1A–1D) and 13 primary hits from the Prestwick Chemical Library (S1A Fig and S1 Table) were initially tested at a 20 μM concentration (Fig 2A–2C). Pol γ (40 nM) alone extended approximately 80% of the substrate by 3–51 nt (Fig 2B, lane 3). While corilagin and carbamazepine from the Zinc database (Fig 2B, molecule no 2–3, respectively), phenolphthalein from KEGG phytochemical compounds (Fig 2B, molecule no 4) and 10 molecules from the Prestwick Chemical Library, acemetacin, indomethacin, amikacin hydrate (Fig 2B, molecule no 5–7, respectively), nicardipine hydrochloride, miconazole, nialamide, nystatin, misoprostol, pimethixene maleate and rifampicin (Fig 2C, molecule no 11–17, respectively) did not significantly affect the strand displacement DNA synthesis activity of Pol γ, three molecules, chenodiol, econazole nitrate, and ketoconazole from the Prestwick Chemical Library (Fig 2B, molecule no 8–10, respectively) decreased the activity of Pol γ about 15.6%, 11.19%, and 15.88%, respectively. We observed that not every small molecule bound to Pol γ protein affected its DNA synthesis activity. This might be because the region of the Pol γ protein interacting with these small molecules did not affect its strand displacement activity. Of the molecules tested, CR showed the greatest inhibition (about 98%) on the strand displacement activity of Pol γ at 20 μM (Fig 2B, lane 4, molecule no 1).

### CR inhibits the DNA synthesis activity of purified Pol γ protein and that of mitochondrial cell extracts

**Fig 3:**
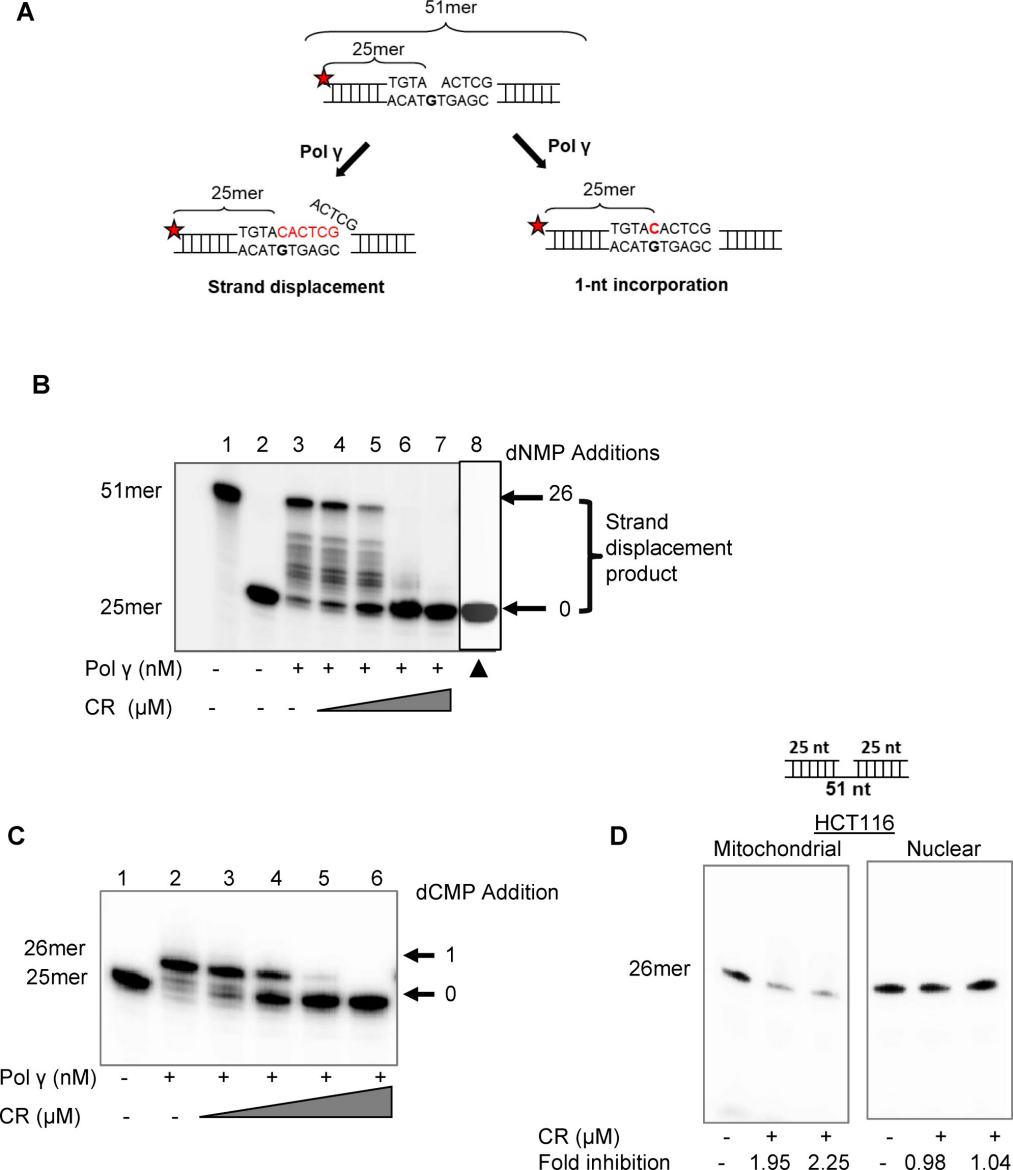
CR inhibits the DNA synthesis activity of purified Pol γ protein and that of mitochondrial cell extracts. (A) Schematic of the ^32^P-labeled (red star) 51 bp DNA substrate containing 1-nt gap at position 26 is shown before and after Pol γ strand displacement and 1-nt incorporation activities (B) Strand displacement reactions contained Pol γ alone (40 nM; lane 3) or together with increasing concentrations of CR (lanes 4–7; 0,5, 1.25, 2.5, and 5 μM). Lane 1, 51mer oligodeoxynucleotide; lane 2, substrate alone. ▲, 40 nM heat-denatured Pol γ protein (lane 8). (C) 1-nt gap incorporation reactions contained Pol γ alone (40 nM; lane 2) or together with increasing concentrations of CR (0,5, 1.25, 2.5, and 5 μM; lanes 3–6, respectively). Lane 1, substrate alone. (D) Single nucleotide DNA synthesis activity in mitochondrial and nuclear extracts treated with/without CR. Treated samples were run in duplicate.

Because CR was the best inhibitor of the strand displacement activity of Pol γ (Fig 2B, molecule no 1), we performed further functional assays with this molecule. We tested the inhibitory effects of increasing concentrations of CR on the strand displacement and 1-nt incorporation activities of Pol γ (Fig 3A). Pol γ (40 nM) extended the majority of substrates (approximately 75%) (Fig 3B, lane 3) and CR (1.25, 2.5, 5 and 10 μM) inhibited the strand displacement DNA synthesis of Pol γ in a concentration dependent manner (Fig 3B, lanes 4–7). Significantly, CR showed almost 94% inhibition of Pol γ strand displacement DNA synthesis at a concentration of 5 μM (Fig 3B, lane 6). To demonstrate that the activity being inhibited was due to Pol γ enzyme rather than a potential impurity, heat-inactivated Pol γ (40 nM) did not alter the size of the substrate (Fig 3B, lane 8). We also assessed the inhibitory activity of increasing concentrations of CR (1.25, 2.5, 5, and 10 μM) on the 1-nucleotide (nt) incorporation activity of Pol γ (Fig 3C, lanes 3–6). Again, a concentration of 5 μM CR inhibited the 1-nt incorporation activity of Pol γ by 95% (Fig 3C, lane 5). Thus, we demonstrate that CR has a strong, concentration-dependent inhibitory effect on both the strand displacement and 1-nt incorporation activities of the Pol γ enzyme.

To evaluate the biological value of CR and assess the specificity to Pol γ, we isolated mitochondria and nucleus from HCT116 cells and determined the effect of CR on 1-nt incorporation DNA synthesis activity (Fig 3D). Samples were assessed for the absence of mitochondrial and nuclear contamination using the mitochondrial and nuclear markers complex IV (COX IV) and Lamin A/C, respectively (S2 Fig). Western blot analysis of mitochondrial and nuclear extracts confirmed the absence of nuclear contamination in the mitochondrial extracts and the absence of mitochondrial contamination in the nuclear extracts (S2 Fig). CR inhibited the 1-nt DNA synthesis activity in the mitochondrial extracts of HCT116 cells around 2-fold, but it did not inhibit the DNA synthesis activity in the nuclear extracts (Fig 3D), indicating the specificity of CR to mitochondrial DNA Pol γ.

Finally, to determine whether the inhibition of DNA synthesis by CR was specific for Pol γ, we also tested the effect of CR on the bacterial *E*. *coli* DNA polymerase Klenow subunit. At 0.02 U Klenow polymerase, 95% of the substrate was converted to strand displacement products (S3 Fig, lane 2). 2.5 and 5 μM CR did not significantly alter the strand displacement activity of the Klenow enzyme (S3 Fig, lanes 3–4). When we increased the concentration of CR to 10 μM, a 30% inhibition of Klenow DNA synthesis was evident (S3 Fig, lane 4). Thus, even at this high concentration, CR could not completely inhibit the Klenow enzyme as it did the Pol γ enzyme. These results attest to the specificity of the CR inhibition of DNA synthesis for Pol γ at lower concentrations.

### CR selectively inhibits the MLH1 deficient cancer cell proliferation

Because the inhibition of Pol γ selectively kills MLH1 deficient cancer cells [3], we tested the effect of four small molecules that came out of our screen, including CR, our top Pol γ inhibitor, on MLH1 deficient colon cancer cell lines. We assessed the effects of Pol γ inhibitor molecules on the cell proliferation of MLH1 deficient HCT116VA and MLH1 proficient HCT116V1 cells in a real-time cell analysis xCELLigence instrument. Western blot analysis confirmed that HCT116VA cells have MLH1 protein but HCT116V1 cells do not have MLH1 protein (S4 Fig).

**Fig 4:**
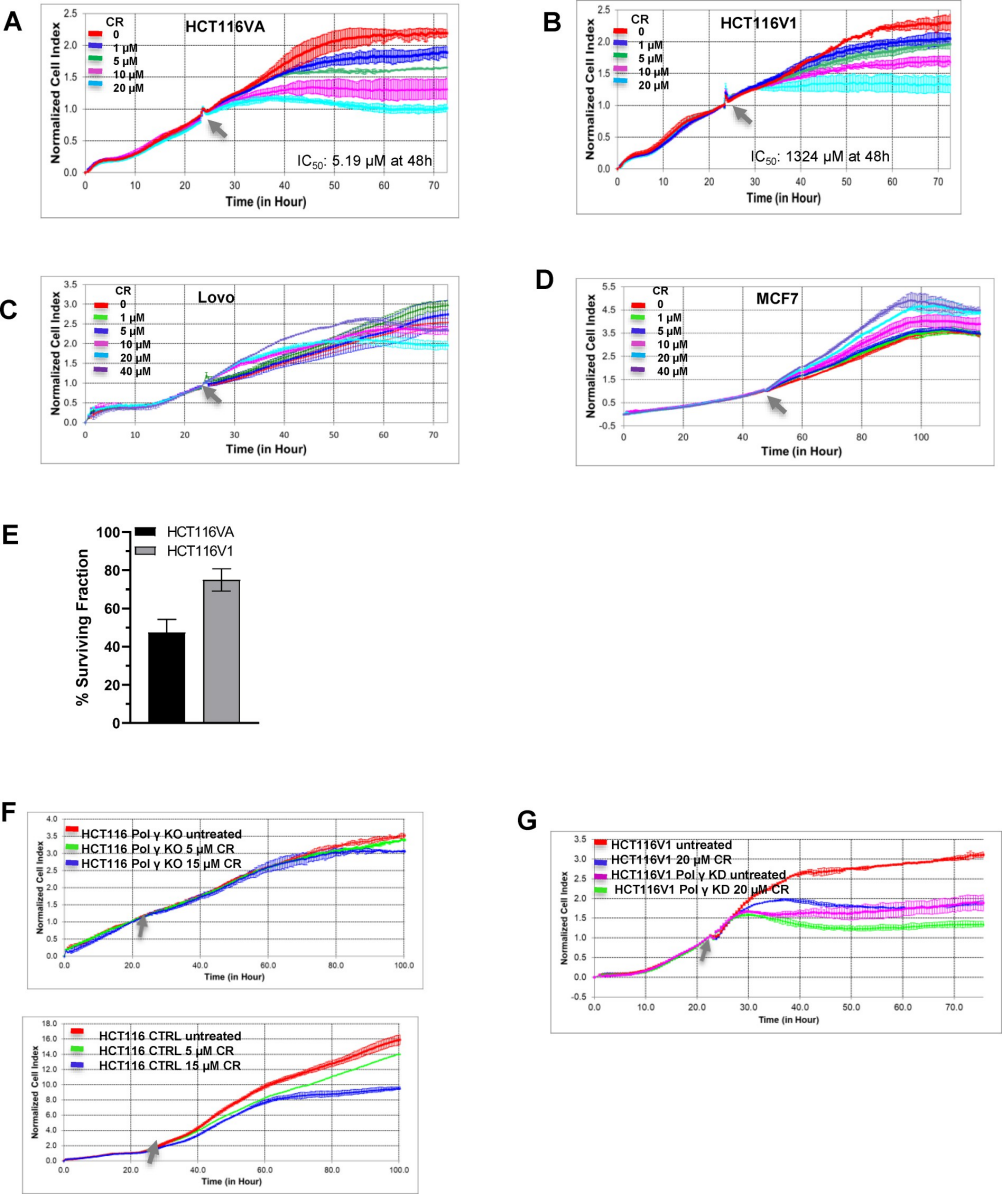
The effects of Pol γ inhibitor molecules on MLH1 deficient and proficient cancer cell proliferation. Real-time dynamic monitoring of the cytotoxic effects of CR on (A) HCT116VA (MLH1 deficient) (B) HCT116V1 (MLH1 proficient) (C) Lovo (MLH1 proficient) (D) MCF7 (MLH1 proficient) cells using the xCELLigence system. Cell growth was continuously monitored every 30 min. Cell index was normalized to the time point of CR administration. Normalized cell index was plotted as the mean value from triplicates; error bars represent the standard deviation of the mean. The Grey arrow indicates the time of CR administration. The xCELLigence RTCA software was used to determine IC_50_ values at 48h post-treatment time point. (E) Effect of 5 μM CR (IC_50_ = 5.19 μM) on colony formation in HCT116VA and HCT116V1 cells. Data are expressed as the mean ± standard deviation of three independent experiments. (F) Upper panel, Real-time dynamic monitoring of the cytotoxic effects of CR on CRISPR/Cas9 Pol γ knockout HCT116 cells and lower panel HCT116 control cells (empty vector control). (G) Real-time dynamic monitoring of the cytotoxic effects of CR on shRNA Pol γ knockdown HCT116V1 cells and HCT116V1 control cells.

Three less potent inhibitors of Pol γ strand displacement DNA synthesis, chenodiol, econazole nitrate, and ketoconazole, did not show the significant effects on the proliferation of either HCT116VA or HCT116V1 cells at 20 μM concentrations (S5A and S5B Fig). On the other hand, 20 μM CR, a potent inhibitor of Pol γ, specifically demonstrated more cytotoxic effects on HCT116VA cells, when compared to HCT116V1 cells (Fig 4A and 4B). In fact, the IC_50_ value of CR on HCT116VA was 5.19 μM, whereas for HCT116V1 this value was 1324 μM at 48h posttreatment (Fig 4A and 4B). Furthermore, CR did not significantly affect the cell proliferation of MLH1 proficient Lovo colon cancer and MCF7 breast cancer cell lines even when concentration was increased to 40 μM (Fig 4C and 4D). These results suggest that CR may inhibit the proliferation of MLH1 deficient colon cancer cells selectively.

We have also tested the clonogenic long-term survival of HCT116VA and HCT116V1 cells after treatment with CR (Fig 4E). Based on the IC50 values at 48 h (Fig 4A), we used 5 μM CR for the clonogenic survival assay. We observed a 53% reduction in the number of surviving HCT116VA colonies and only a 25% reduction in HCT116V1 colonies as a consequence of exposure to 5 μM CR (Fig 4E). Thus, MLH1-deficient HCT116VA cells were more sensitive to CR than MLH1-proficient HCT116V1 cells, both in a short-term real-time dose-response survival assay (Fig 4A and 4B) and in a long-term colony formation assay (Fig 4E).

To determine if the antiproliferative effect of CR was mediated through inhibition of Pol γ, we compared its effect on Pol γ knockout HCT116 cells. To this end, we generated POLGA gene knockout HCT116 cells using CRISPR/Cas9 genome editing. The knockout percentage of Pol γ of HCT116-CRISPR#6 clone # 43 is 99.16% (S6A Fig, lane 4). These single-cell clones were selected for further characterization. Extensive Sanger sequencing of exon 2, where CRISPR/Cas9 targets, showed that clone # 43 had frameshift mutations within the open reading frame on both alleles, which resulted in premature stop codons (S6B Fig), and this clone was used in cellular experiments (Fig 4F). CR treatment (5 μM and 15 μM) for 96 h did not affect the cell proliferation of Pol γ knockout HCT116 cells (Fig 4F, upper panel) whereas it inhibited the cell proliferation of HCT116 control cells (empty vector control) (Fig 4F, lower panel). shRNA knockdown of Pol γ in MLH1 proficient HCT116V1 cells also showed that the effects of CR (20 μM) treatment on the cell proliferation of HCT116V1 cells was much higher than that of Pol γ shRNA knockdown HCT116V1 cells (Fig 4G). The knockdown percentage of Pol γ of HCT116V1 cells is 87.2% (S6C Fig). In this experiment, we used the CR concentration (20 μM) that affects the cell proliferation of HCT116V1 (Fig 4B) to see its effects on Pol γ shRNA knockdown HCT116V1 cells and control cells. Thus, these results suggest that the effects of CR could be Pol γ dependent.

### CR increases ROS and 8-OHdG levels of mtDNA and decreases mtDNA copy number

**Fig 5:**
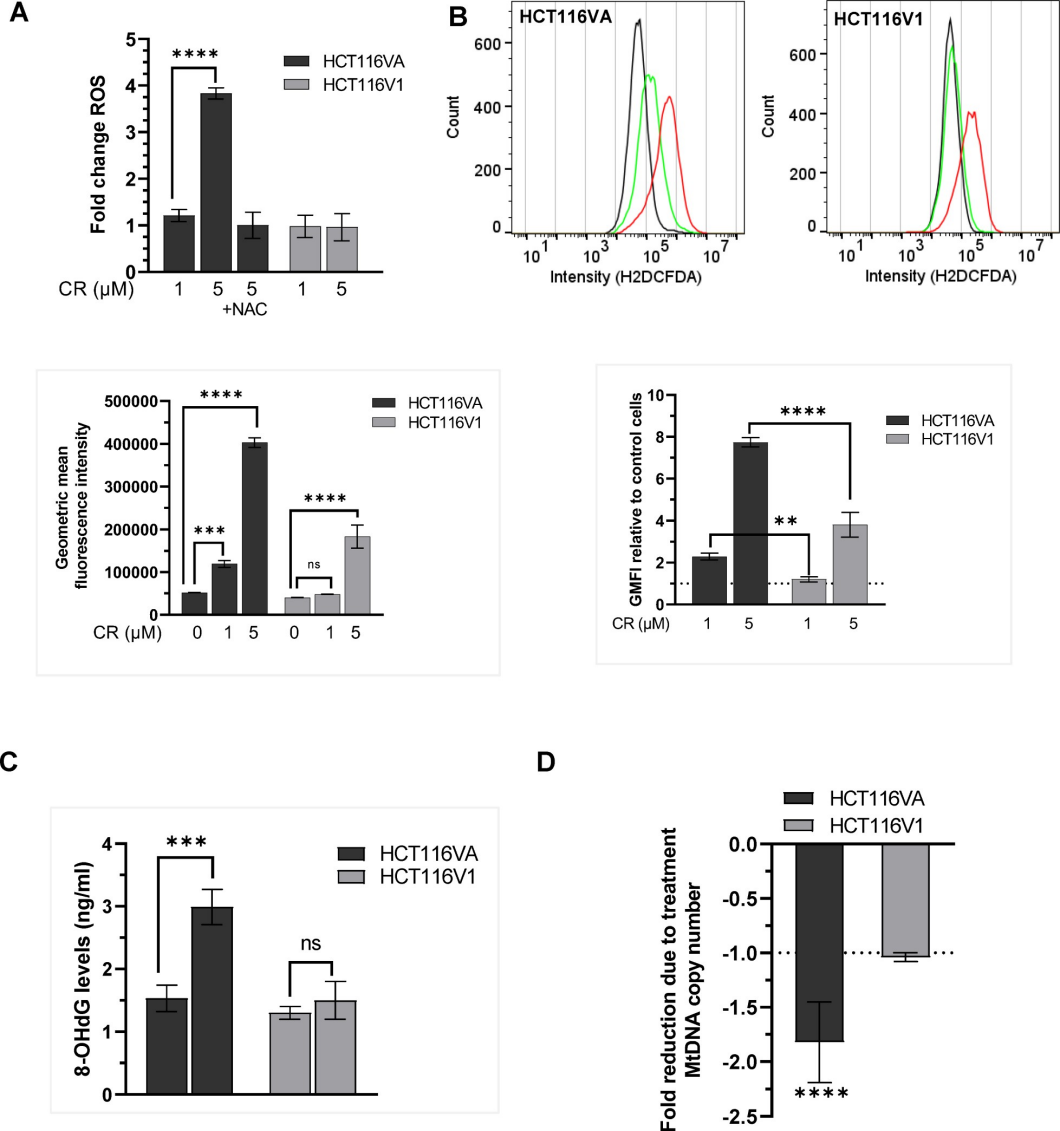
CR increases ROS and decreases the mtDNA copy number in MLH1 deficient cells. (A) ROS measured using the H2DCFDA assay in HCT116VA and HCT116V1 cells treated with CR for 48h and normalized to the corresponding viable cells. Data are shown as mean fold change to untreated control ± STD. (B) Flow cytometry measurement of ROS levels by H2DCFDA fluorescence assay. Plots of the fluorescence intensities of the DCF dye in cells exposed to 1 μM CR (green color), 5 μM CR (red color), or untreated cells (black color) for 1 h. Data are given as the ratio of geometric mean fluorescence intensity (GMFI) and GMFI of treated relative to untreated cells (control). (C) The quantification of 8-OHdG levels in HCT116VA and HCT116V1 cells upon treatment of CR. (D) MtDNA copy number. The fold change in mtDNA copy number due to treatment was calculated by 2^-ΔΔCt^ method. Each experiment was performed in triplicate. **p = 0.002; ***p = 0.0003; ****p< 0.0001; ns, not significant.

Because ROS induces oxidative DNA damage repaired by BER [36], we tested if CR induces ROS production in HCT116VA and HCT116V1 cells. We measured the intracellular ROS levels using dichlorodihydrofluorescein diacetate (DCFDA), which is oxidized to highly fluorescent dichlorofluorescein (DCF) in the presence of ROS. We normalized the fluorescence data to the corresponding cell viability data at 48 h CR incubation because of the observed cell death at the 48 h post-treatment point (Fig 5A, 48 h). CR increased ROS production in MLH1 deficient HCT116VA cells but not in MLH1 proficient HCT116V1 cells (Fig 5A). The treatment of cells with ROS scavengerNAC together with CR did not cause a change in ROS levels indicating that an increase in ROS in the cells was due to CR treatment (Fig 5A).

We also measured the short-term effects of CR (1 h) on ROS levels by flow cytometry (Fig 5B). Both 1 μM and 5 μM CR caused a statistically significant (p< 0.05) increase in ROS levels of HCT116VA cells compared with untreated controls whereas 1 μM CR did not affect the generation of ROS in MLH1 positive HCT116V1 cells. The baseline ROS levels (Fig 5B, flow cytometric histograms, black color, untreated) in HCT116VA and HCT116V1 cells were similar. CR treatment caused ROS induction in HCT116VA cells (Fig 5B, bar graph, left panel). The increase in ROS levels following 5 μM CR treatment was around 2-fold higher in HCT116VA cells (7.74±0.22-fold) compared to HCT116V1 cells (3.81±0.59-fold) (Fig 5B, bar graph, right panel). These data suggest that CR may cause a higher ROS production in MLH1 deficient HCT116VA cells compared with MLH1 dependent HCT116 cells, and the selectivity may depend on CR dose.

To further investigate if the increase in ROS production following CR treatment causes oxidative mtDNA damage, we measured the 8-OHdG levels of mtDNA. As shown in Fig 5C, CR increased 8-OHdG concentration in mtDNA of HCT116VA cells by 1.95-fold (2.99±0.28 ng/ml) whereas it did not cause a statistically significant increase in 8-OHdG levels of mtDNA in HCT116V1 cells (p˃0.05). We tested the effects of CR on mtDNA copy number and detected a significant decrease (p<0.05) in mtDNA copy number when compared to its controls in HCT116VA cells but not in HCT116V1 cells (Fig 5D). We observed a decrease in mtDNA copy number following 24 h CR treatment, but not following 8h CR treatment (S7 Fig). As shown before, HCT116 cells have a lower mtDNA copy number compared to MLH1 positive HCT116 cells [37]. S7 Fig also shows that untreated HCT116VA cells have a lower mtDNA copy number compared with untreated HCT116V1 cells. CRISPR knockout of Pol γ was associated with around 41.2% reduced mtDNA copy number (S7 Fig). Treatment of the Pol γ knockout HCT116 cells with CR for 24 h caused a 1.5-fold decrease in mtDNA copy number, but this decrease was not statistically significant (p = 0.12).

### CR suppresses HCT116 xenograft tumor growth, but not HCT116V1 and Lovo xenograft tumor growth

**Fig 6:**
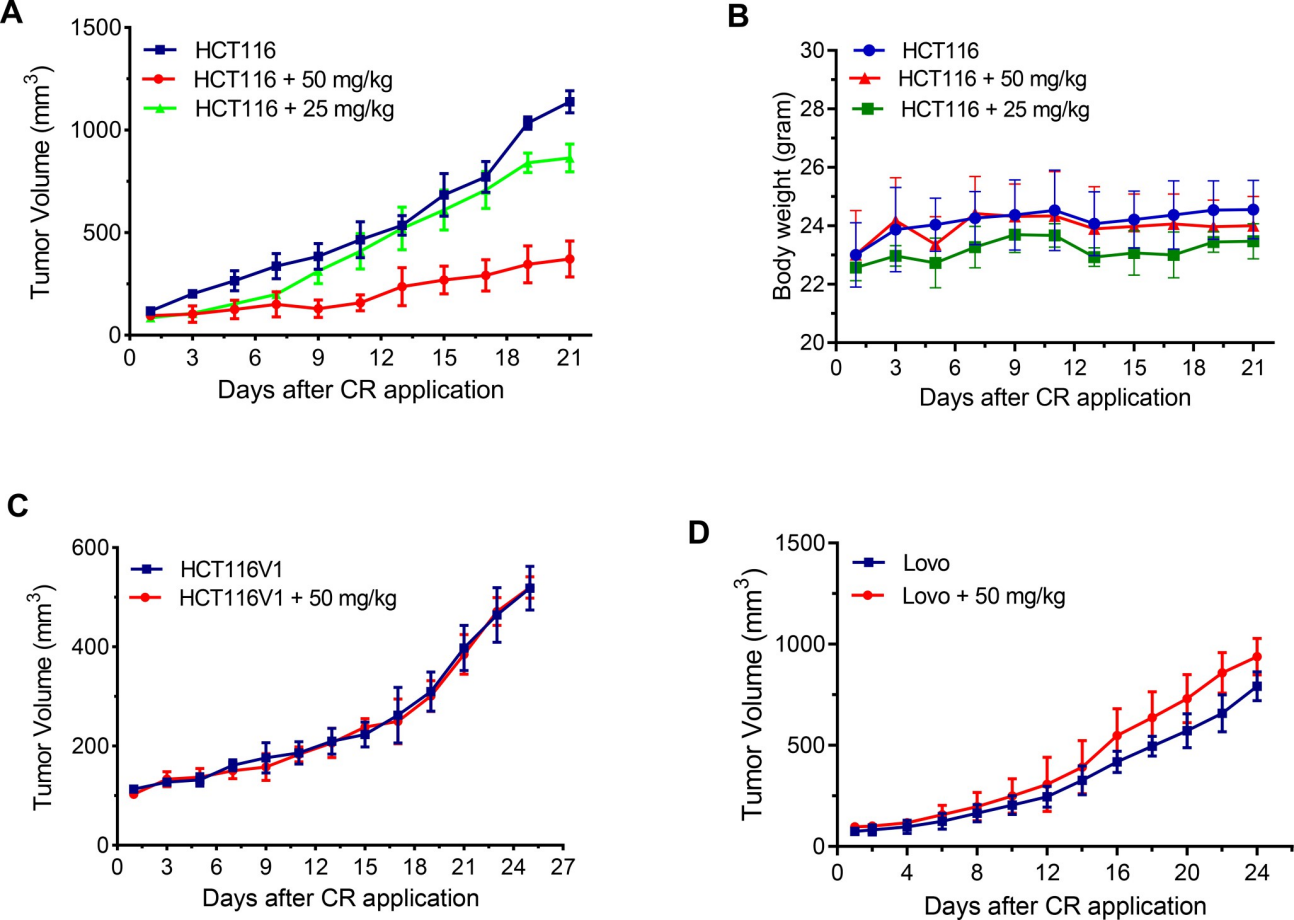
CR suppresses the growth of HCT116 xenograft tumors. (A) Growth curves of HCT116 xenograft tumors (n = 6, tumor volume mm^3^) treated with CR and 0.02% DMSO (n = 6, control). p < 0.01, 50 mg/kg compared with control group; p˃0.05, 25 mg/kg compared with control group. (B) The corresponding body weight changes during the treatment. p˃0.05, compared with a control group. (C) Growth curves of HCT116V1 xenograft tumors (n = 3, tumor volume mm^3^) treated with CR and 0.02% DMSO (n = 3, control). p˃0.05, compared with a control group. (D) Growth curves of Lovo xenograft tumors (n = 5, tumor volume mm^3^) treated with CR and 0.02% DMSO (n = 5, control). p˃0.05, compared with a control group.

To verify our *in vitro* cellular findings that CR selectively inhibits the proliferation of MLH1 deficient HCT116 cells, we established HCT116, HCT116V1, and Lovo xenograft tumor models in nude mice. CR at 25 and 50 mg/kg dose per day showed significant HCT116 xenograft tumor growth inhibition compared to vehicle alone controls (Fig 6A). Three weeks of CR administration (50 mg/kg) caused a 67.33% decrease in tumor volume as compared with the control group (Fig 6A; p < 0.01). The lack of significant body weight loss compared with control animals demonstrated that both doses were well tolerated and were not toxic (Fig 6B). Significantly, CR did not affect the tumor growth of HCT116V1 (Fig 6C) and Lovo xenografts (Fig 6D). These findings demonstrate that CR is a specific inhibitor of the growth of MLH1 deficient HCT116 xenograft tumors, but not MLH1 proficient HCT116V1 and Lovo xenograft tumors *in vivo*.

## Discussion

The desired approach for successful cancer therapy is to selectively kill cancer cells without damaging normal cells. Because of the inhibition of Pol γ in its synthetic lethal partner gene, MLH1 deficient cancer cells could provide this selectivity [3] and because known inhibitors of Pol γ have not been used for clinical treatment of MLH1 deficient cancers, we searched for new Pol γ inhibitor molecules that are selectively cytotoxic to MLH1 deficient cancer cells. The powerful Pol γ inhibitor identified in this study is CR that binds to the Pol γ enzyme with high affinity, specifically inhibits the activity of this enzyme, and can slow the *in vivo* growth of MLH1 deficient cancer cells and tumor xenografts in a synthetic lethal manner.

Cell viability-based small molecule screening studies reported that 2-benzoyl-6-(2,3-dimethoxybenzylidene)-cyclohexanol (AS13) is selectively toxic to MLH1 deficient cancer cells [38] and that menadione and cytosine-based nucleoside analogs such as cytarabine are selectively lethal to both MLH1 and MSH2 deficient cancer cells [3, 21, 22]. The selective cytotoxicity of these compounds was associated with increased levels of cellular oxidative stress, and thus oxidative DNA damage and cell death [21, 38]. This is supported by a recent study showing that MLH1 deficient HCT116 cancer cells have reduced antioxidant response, increased ROS, and are more vulnerable to DNA damage due to their perturbed mitochondrial metabolism [37]. Consistent with these findings, we showed that CR caused an increase in ROS production and 8-OHdG levels of mtDNA, and a decrease in mtDNA copy number in MLH1 deficient HCT116 cells, but not in MLH1 proficient HCT116 cells. The molecular basis of oxidative stress-induced selective cell death in these cell lines may differ from each other. This study suggests that CR may cause mitochondrial dysfunction and cell death by inhibiting Pol γ activity and oxidative mtDNA damage repair, and incomplete repair of higher oxidative DNA damage in MLH1 deficient cells may induce selective cell death.

The azo class dyes used in textile, printing, chemical, food, and drug industries, including CR may have toxic, mutagenic, and genotoxic effects [39]. On the other hand, CR has beneficial characteristics as it inhibits amyloid oligomerization and fibril formation, and prevents the accumulation and toxicity of proteins aggregates in neurodegenerative diseases, for example, Alzheimer’s disease, Huntington’s disease (HD), and spinocerebellar ataxia type 14 [40, 41]. Sanchez et al showed that CR ameliorates symptoms of disease in HD mice and extended its life span at a dose of 1 mg/30 g administered every 48 h intraperitoneally for 28 days [42]. In our study, CR at 50 mg/kg (1.5 mg/30 g) injected intraperitoneally for 21 days showed the synthetic lethal antitumor effects and did not cause a weight loss. It has also been shown that CR has virucidal effects through inhibiting RNA/DNA polymerase. Several cellular and animal studies have shown that the lethal dose of CR changes according to cell, tissue, and animal types, thereby using CR at optimum low dosage would reduce its side effects. For example, the lethal dose of CR in rat was estimated to be 190 mg/kg and administration of 400 mg/kg CR was lethal for pregnant rats [40].

The toxic side effects of CR could be overcome and the efficacy should be increased by targeting CR to mitochondria. Although the synthetic lethal relationship between Pol γ and MLH1 leads to the selective killing of MLH1 deficient cancer cells by showing minimal cytotoxicity to MLH1 proficient cells, transporting CR to mitochondria with nanocarrier systems will likely improve its stability and therapeutic effect, reduce off-target effects and decrease its toxicity. Thus, it can be concluded that further evaluation of CR and its derivatives would render it a promising therapeutic agent for the MLH1 deficient cancers’ therapy.

## Supporting information

S1 Raw images (PDF)

S1 Table Relative binding levels of prestwick chemical library molecules to Pol γ. (PDF)

S1 Fig Screening of small molecules for direct binding to Pol γ. **A.** Screening of Prestwick Chemical Library molecules for direct binding to Pol γ. The data points represented with circles and red circles represent the primary hits with relatively high specific binding for Pol γ protein. Molecules that bound Pol γ more than an arbitrary threshold of 3-fold compared to the negative control protein (RNA helicase A) were designated as primary hits and indicated as red dots in sensorgrams. **B.** Binding kinetics of fialuridine (positive control) to Pol γ. All data are presented as the means ± SD of three independent experiments. The chemical structure of fialuridine is given.
(TIF)

S2 Fig Western blot analysis of mitochondrial and nuclear extracts isolated from HCT116 cells confirmed the absence of nuclear contamination in the mitochondrial extracts and the absence of mitochondrial contamination in the nuclear extracts. (TIF)

S3 Fig Klenow activity. Lane 1, substrate alone. Lane 2, Klenow (0.02 U/μl) alone and lanes 3–5 are Klenow (0.02 U/μl) with increasing concentrations of CR (1.25, 2.5, and 5 μM).
(TIF)

S4 Fig MLH1 levels of HCT116 cell lines were determined by Western blotting. β-actin is used as a loading control. M, Molecular marker-Precision plus protein dual color standards from Bio-Rad.
(TIF)

S5 Fig The effects of econazole nitrate, chenodiol, and ketoconazole on MLH1 deficient and proficient cancer cell proliferation. Real-time dynamic monitoring of the cytotoxic effects of econazole nitrate, chenodiol, and ketoconazole on **A.** HCT116VA (MLH1 deficient), **B**. HCT116V1 (MLH1 proficient) cells using the xCELLigence system. Cell growth was continuously monitored every 30 min. Cell index was normalized to the time point of compound administration. Normalized cell index was plotted as the mean value from triplicates; error bars represent the standard deviation of the mean. The Grey arrow indicates the time of the compound administration.
(TIF)

S6 Fig Generation of Pol γ knockout HCT116 cell lines by CRISPR/Cas9. **A.** Western blot analysis with HCT116 single cell clones (Lane 1. Parental HCT116, lane 2. HCT116-CRISPR#1 clone #12, lane 3. HCT116-CRISPR#6 clone #27, lane 4. HCT116-CRISPR#6 clone #43, lane 5. HCT116-CRISPR#6 clone #44. M, Molecular marker-Precision plus protein dual color standards from Bio-Rad. **B.** Sanger sequencing results of HCT116 clones. The translation start codon is shown in bold, the HpyCH4III restriction site is shown in italics, and the CRISPR/Cas9 sgRNA target site is underlined. The dashed lines indicates deletions.
(TIF)

S7 Fig CR decreases mtDNA copy number in MLH1 deficient cells. *p = 0.0004; **p = 0.0001; ***p< 0.0001; ns, not significant.
(TIF)
