# Supplementary material for: Targeting mitochondrial DNA polymerase gamma for selective inhibition of MLH1 deficient colon cancer growth
Source: PLoS One. 2022 Jun 3;17(6):e0268391. doi: 10.1371/journal.pone.0268391 (PMC9165880; doi:10.1371/journal.pone.0268391)

Fig 2B

PhosphorImager (Typhoon FLA9500)

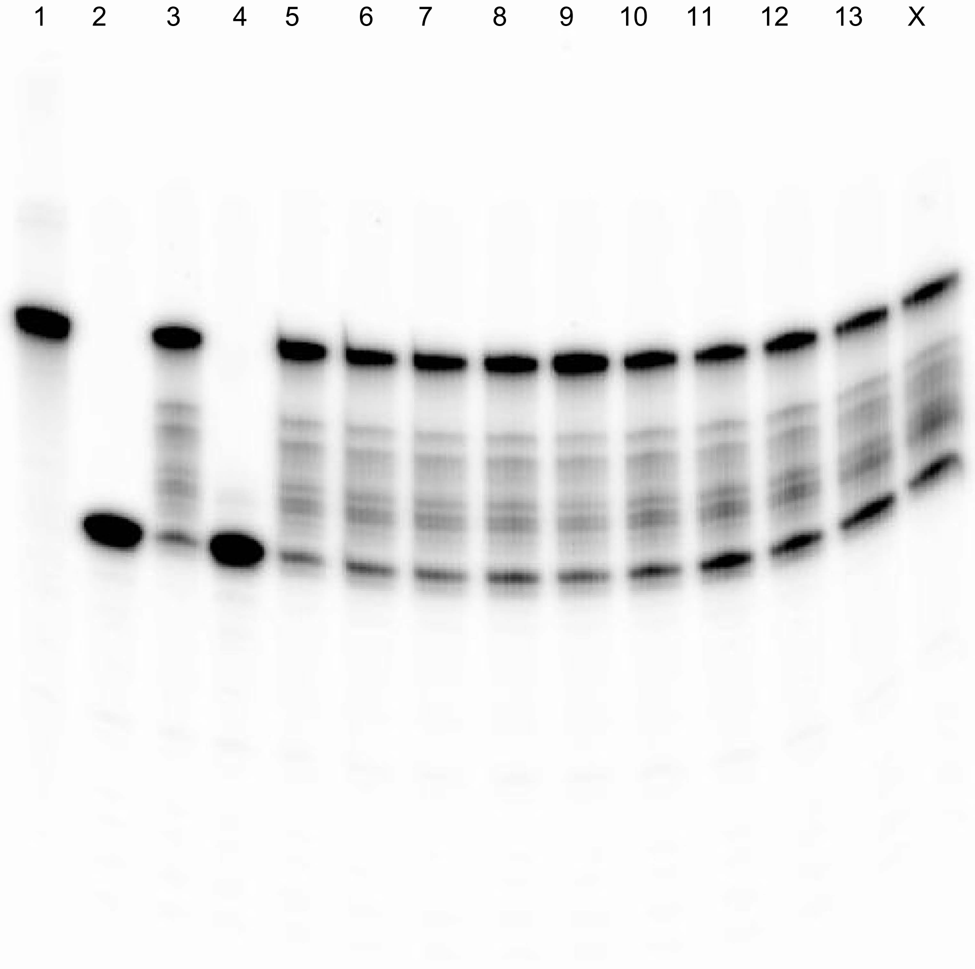

Fig 2C

PhosphorImager (Typhoon FLA9500)

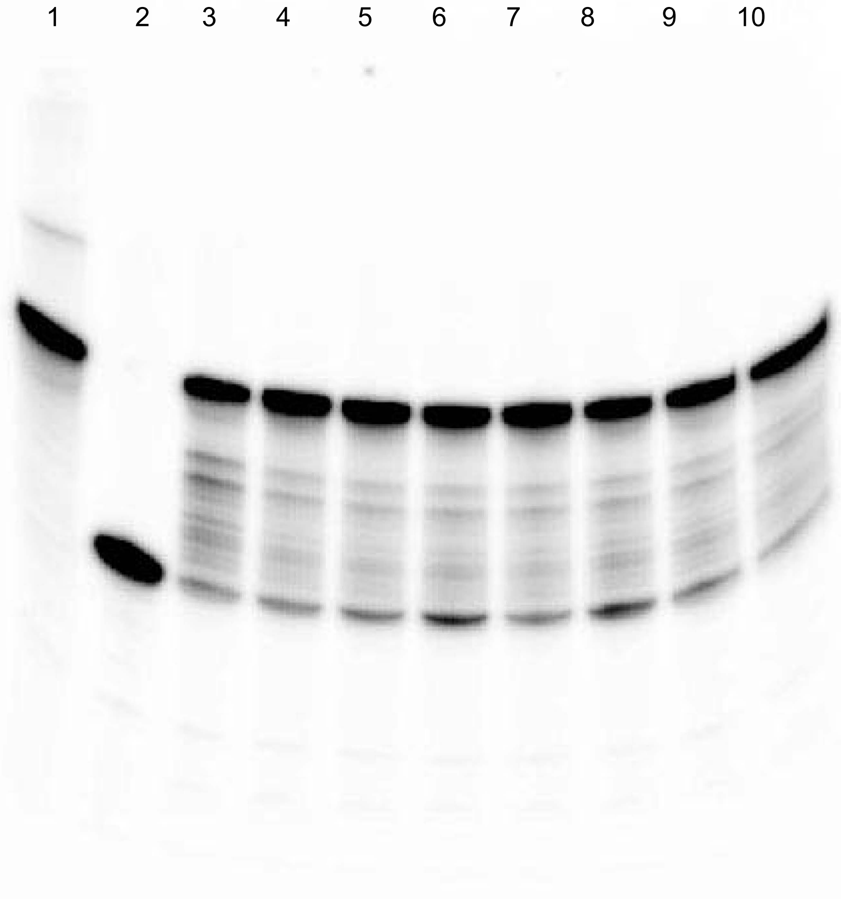

Fig 3B

PhosphorImager (Typhoon FLA9500)

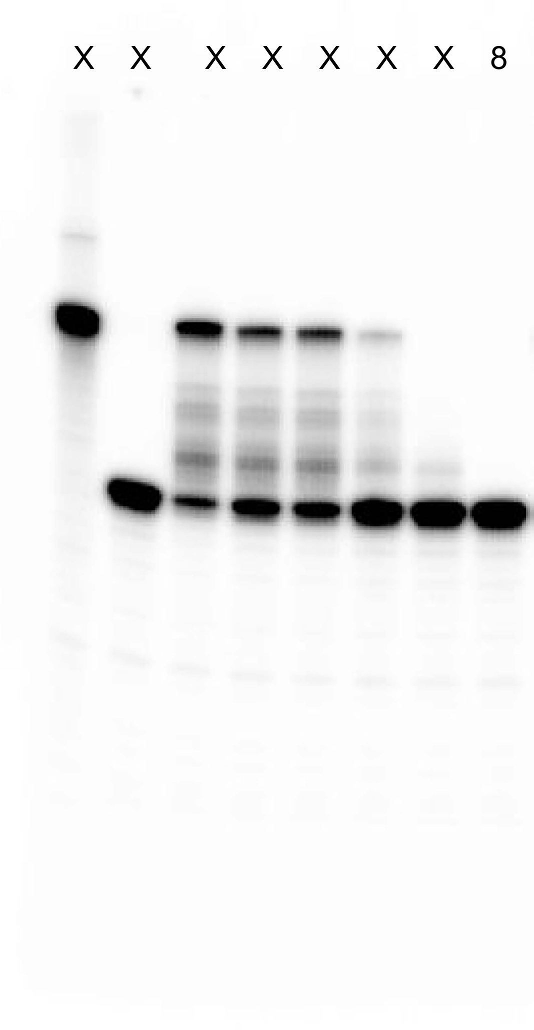

Fig 3B and S3 Fig

PhosphorImager (Typhoon FLA9500)

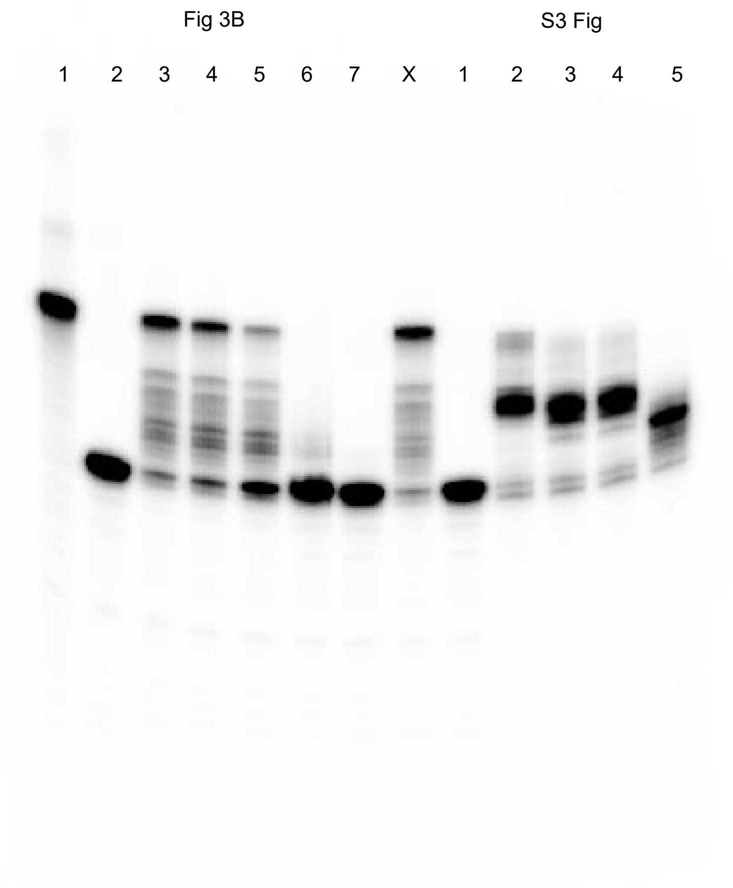

Fig 3C

PhosphorImager (Typhoon FLA9500)

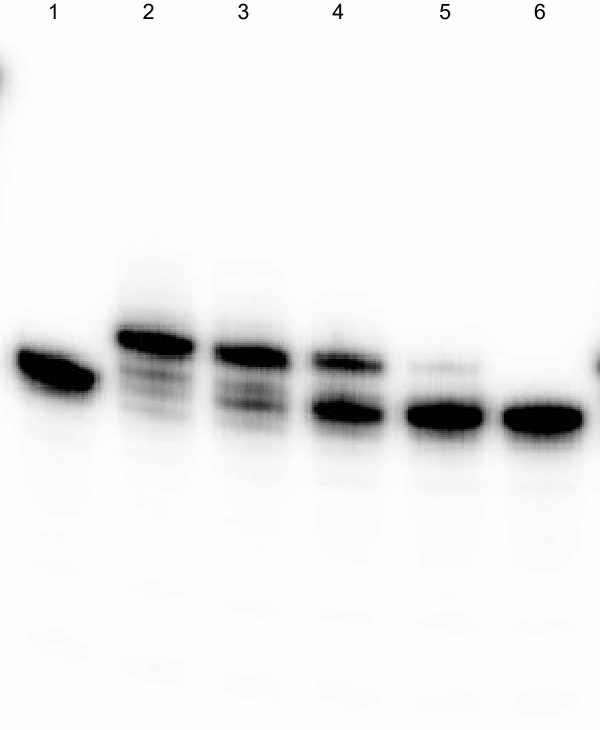

Fig 3D

PhosphorImager (Typhoon FLA9500)

Mitochondrial Nuclear

1 2 3 X X X 1 2 3 X X X

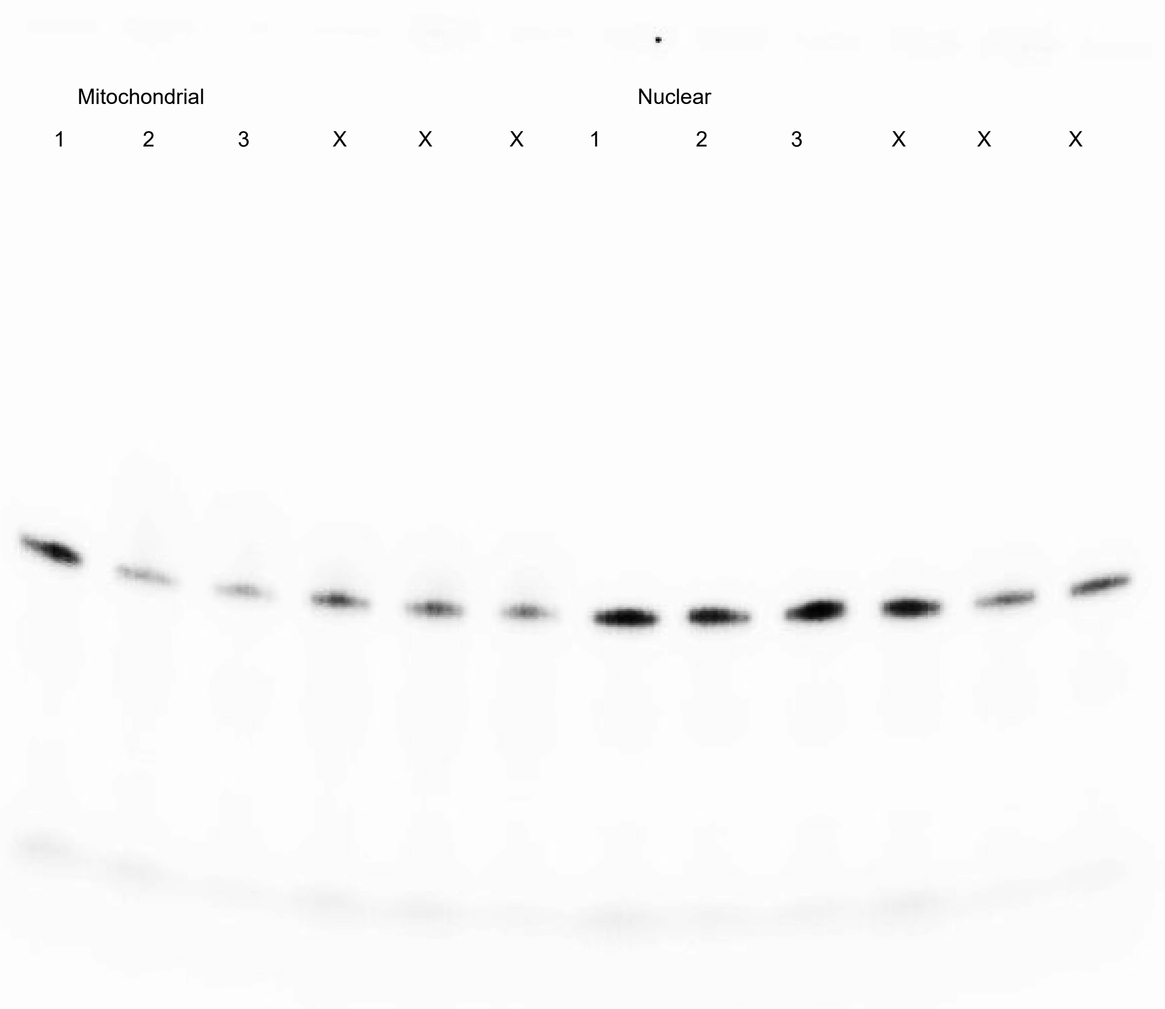

S2 Fig
BioRad Image Lab

Top Panel Lamin A/C

Mitochondrial Nuclear

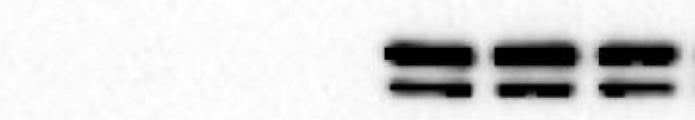

Mitochondrial Nuclear

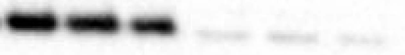

S2 Fig
BioRad Image Lab
Lower Panel, COXIV

S4 Fig
BioRad Image Lab
Upper Panel, MLH1

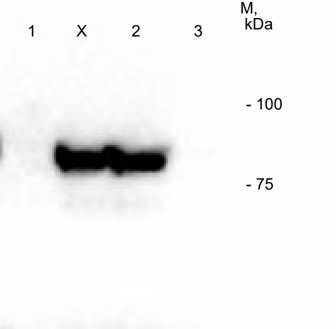

S4 Fig
BioRad Image Lab
Lower Panel, B-Actin

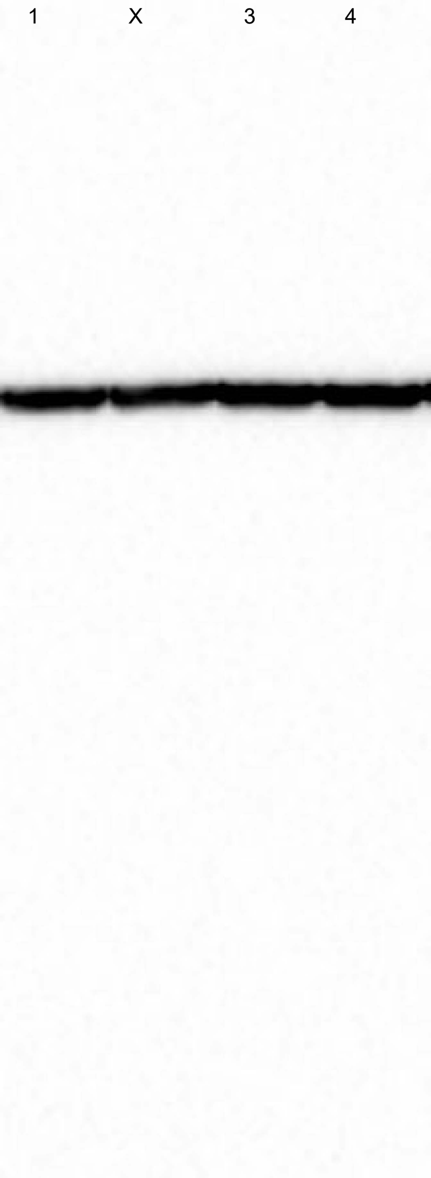

S6A Fig
BioRad Image Lab
Upper Panel, Polg

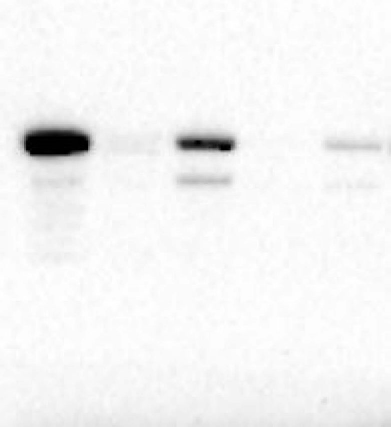

S6A Fig
BioRad Image Lab
Lower Panel, B-Actin

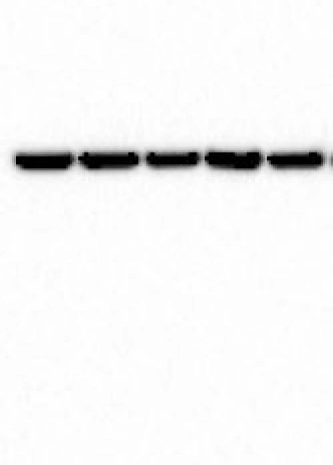

S6C Fig
BioRad Image Lab
Upper Panel, Polg

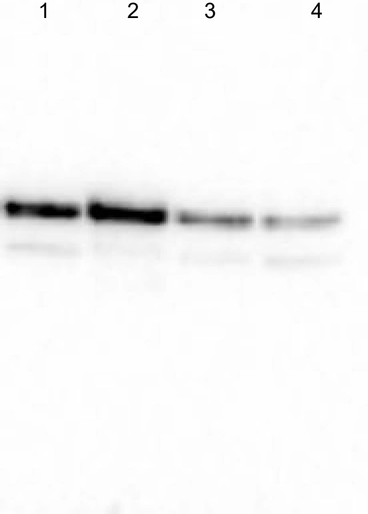

S6C Fig
BioRad Image Lab
Lower Panel, B-Actin

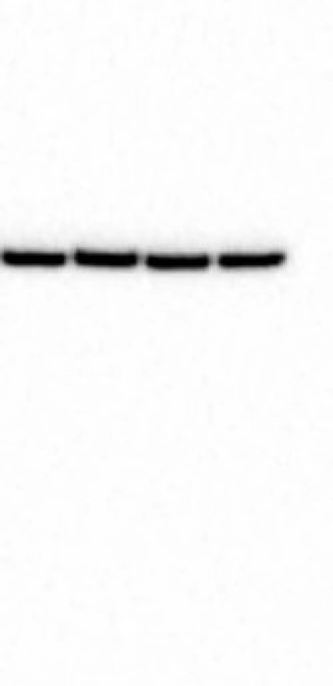

Supplement: S1 Raw images — (PDF) [file pone.0268391.s001.pdf]
