# Supplementary material for: Targeting mitochondrial DNA polymerase gamma for selective inhibition of MLH1 deficient colon cancer growth
Source: PLoS One. 2022 Jun 3;17(6):e0268391. doi: 10.1371/journal.pone.0268391 (PMC9165880; doi:10.1371/journal.pone.0268391)
Supplement: S1 Table — (PDF) [file pone.0268391.s002.pdf]

S Table. Relative binding levels of Prestwick Chemical Library Molecules to Pol γ

Chemical name	Molecular weight (Da)	Relative Binding (RU)	Rmax%
Nicardipine hydrochloride	516.00	21.57227	113
Amikacin hydrate	621.64	20.72363	91
Miconazole	416.4	18.50781	120
Nialamide	298.35	17.48828	159
Nystatine	926.12	17.11621	50
Econazole nitrate	444.70	17.90137	109
Ketoconazole	531.44	17.07031	87
Misoprostol	382.55	15.13281	107
Pimethixene maleate	409.51	14.00195	92
Acemetacin	415.83	13.0127	85
Indomethacin	357.80	11.68359	89
Chenodiol	392.58	10.52051	71
Rifampicin	822.96	10.2627	33
