# Supplementary figures and images for: Targeting mitochondrial DNA polymerase gamma for selective inhibition of MLH1 deficient colon cancer growth
Source: PLoS One. 2022 Jun 3;17(6):e0268391. doi: 10.1371/journal.pone.0268391 (PMC9165880; doi:10.1371/journal.pone.0268391)

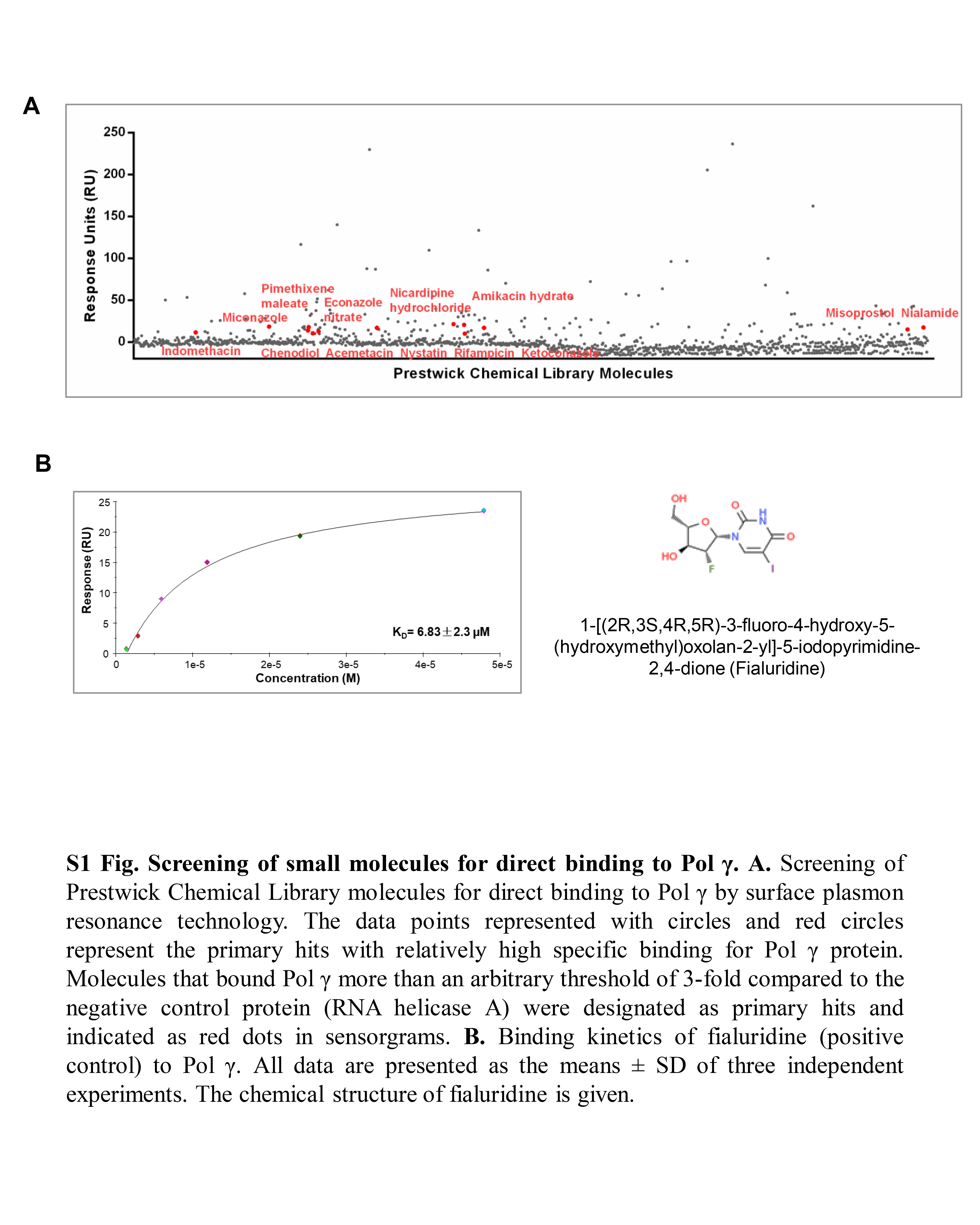

Supplement: S1 Fig — A. Screening of Prestwick Chemical Library molecules for direct binding to Pol γ. The data points represented with circles and red circles represent the primary hits with relatively high specific binding for Pol γ protein. Molecules that bound Pol γ more than an arbitrary threshold of 3-fold compared to the negative control protein (RNA helicase A) were designated as primary hits and indicated as red dots in sensorgrams. B. Binding kinetics of fialuridine (positive control) to Pol γ. All data are presented as the means ± SD of three independent experiments. The chemical structure of fialuridine is given. (TIF) [file pone.0268391.s003.tif]

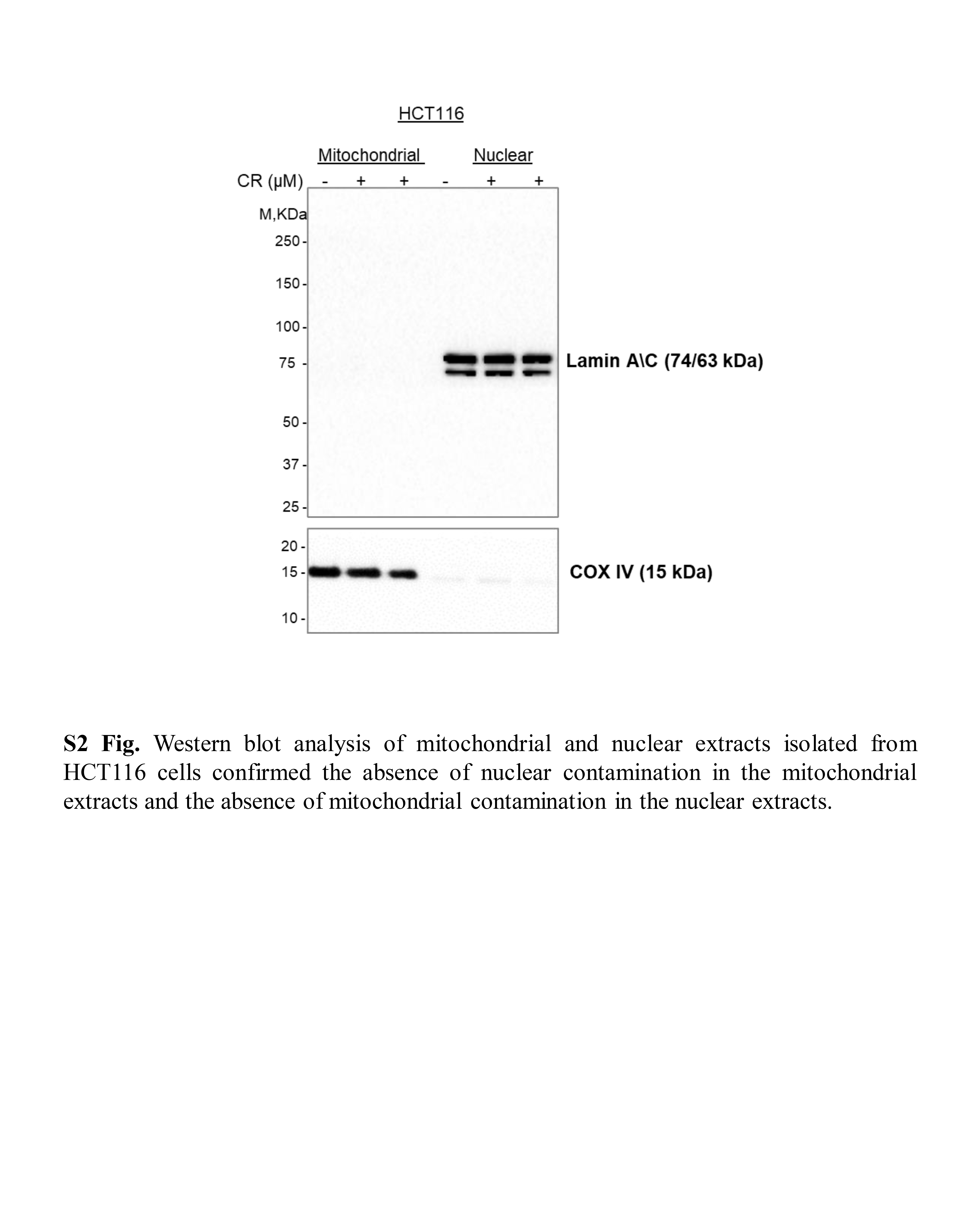

Supplement: S2 Fig — (TIF) [file pone.0268391.s004.tif]

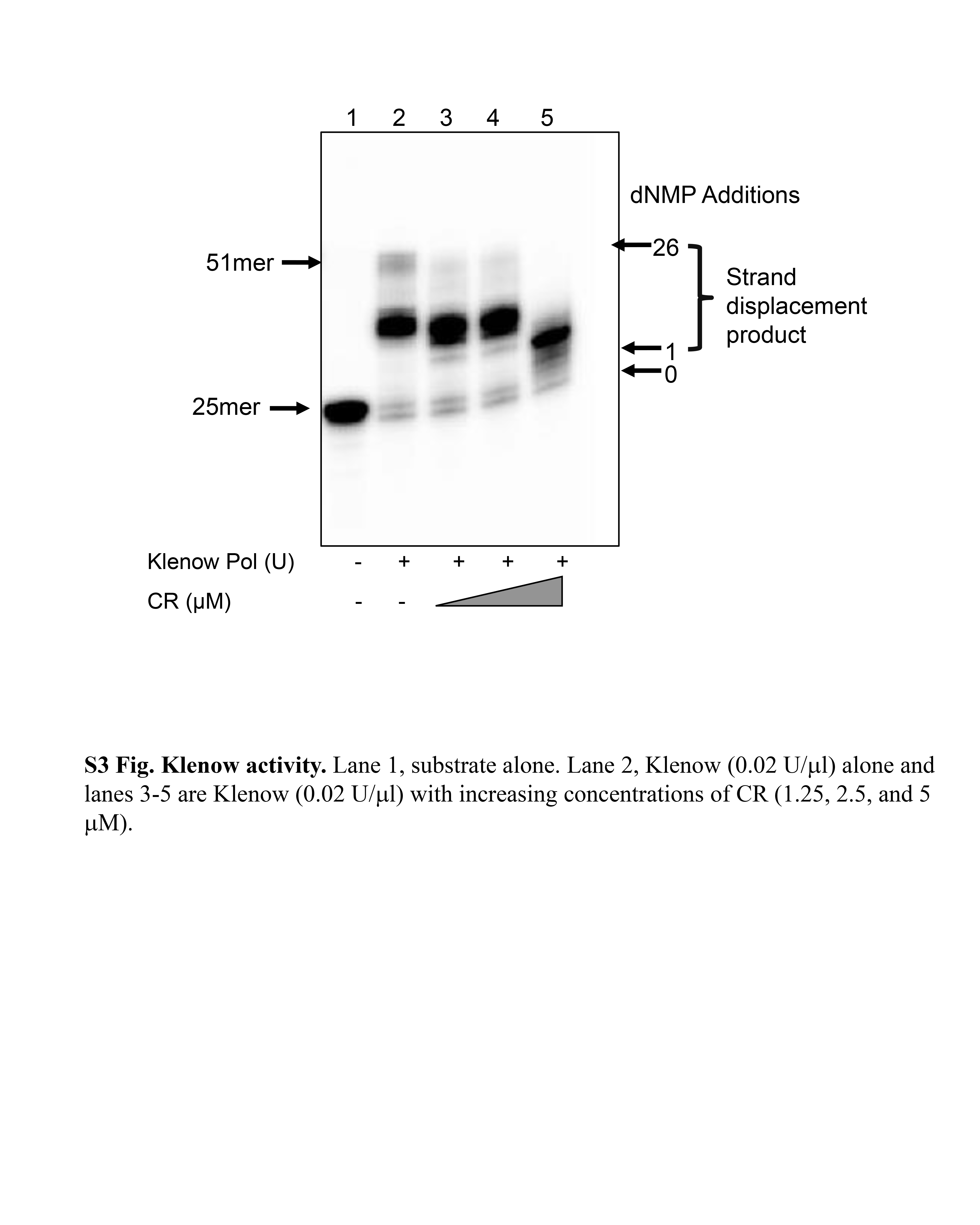

Supplement: S3 Fig — Lane 1, substrate alone. Lane 2, Klenow (0.02 U/μl) alone and lanes 3–5 are Klenow (0.02 U/μl) with increasing concentrations of CR (1.25, 2.5, and 5 μM). (TIF) [file pone.0268391.s005.tif]

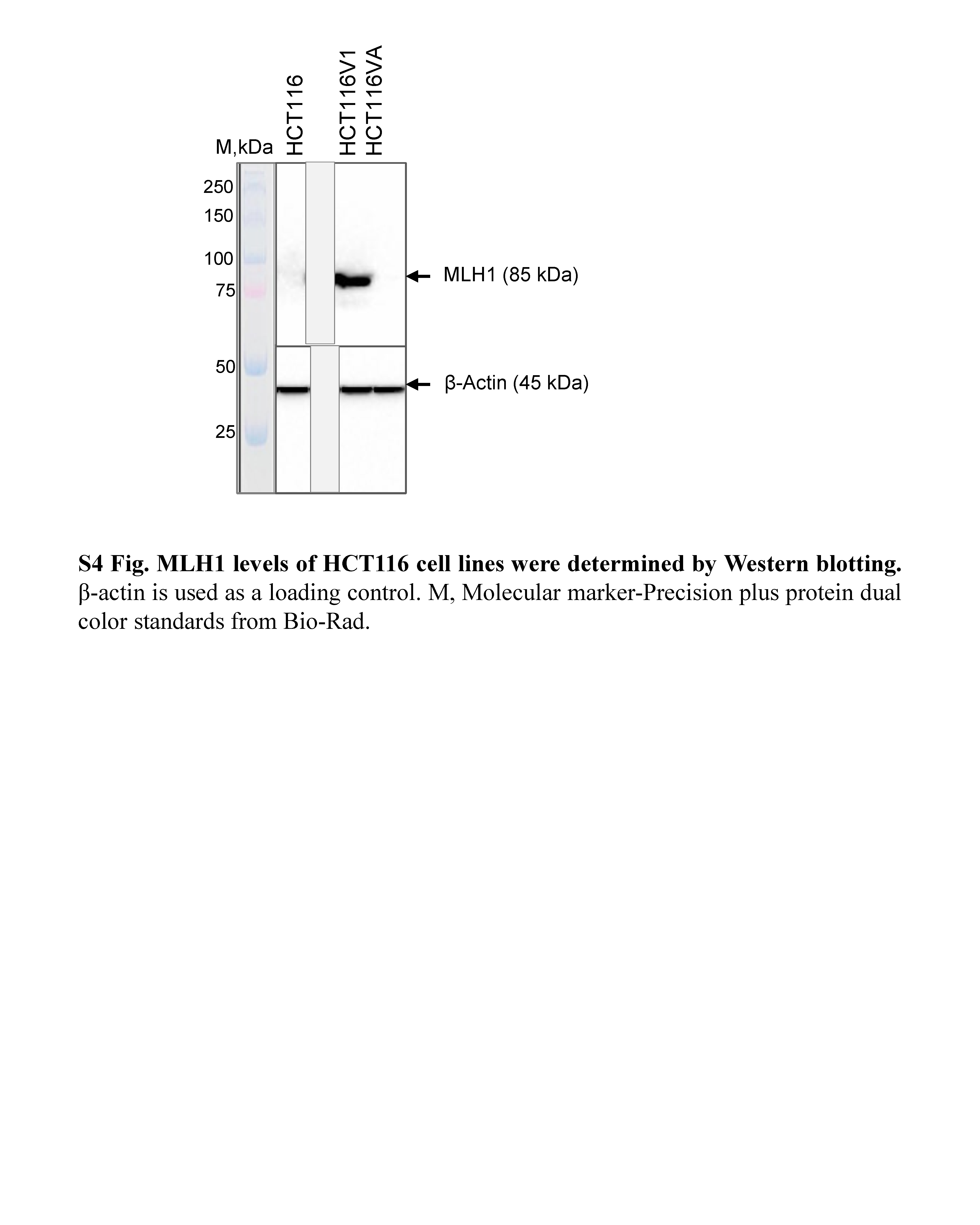

Supplement: S4 Fig — β-actin is used as a loading control. M, Molecular marker-Precision plus protein dual color standards from Bio-Rad. (TIF) [file pone.0268391.s006.tif]

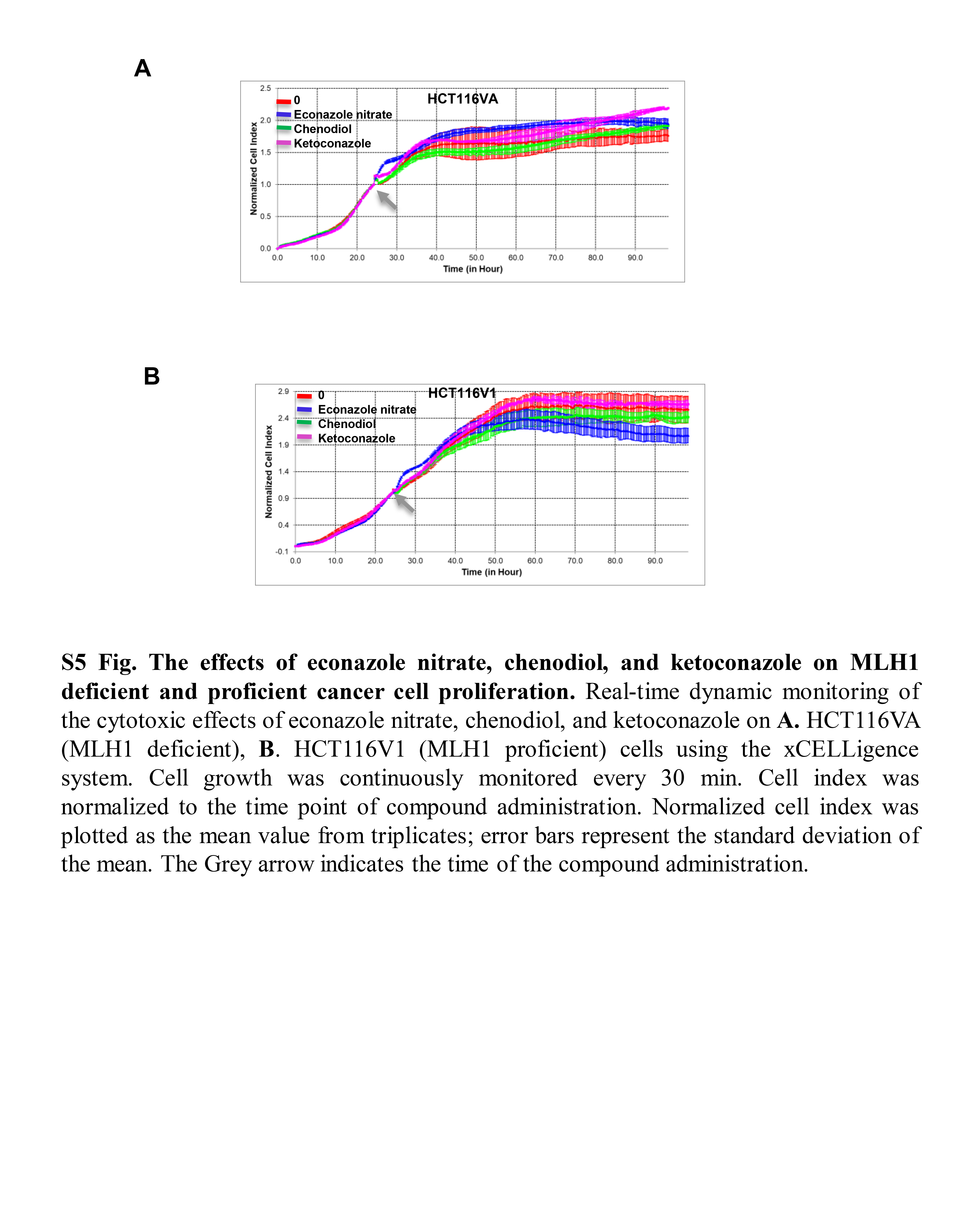

Supplement: S5 Fig — Real-time dynamic monitoring of the cytotoxic effects of econazole nitrate, chenodiol, and ketoconazole on A. HCT116VA (MLH1 deficient), B. HCT116V1 (MLH1 proficient) cells using the xCELLigence system. Cell growth was continuously monitored every 30 min. Cell index was normalized to the time point of compound administration. Normalized cell index was plotted as the mean value from triplicates; error bars represent the standard deviation of the mean. The Grey arrow indicates the time of the compound administration. (TIF) [file pone.0268391.s007.tif]

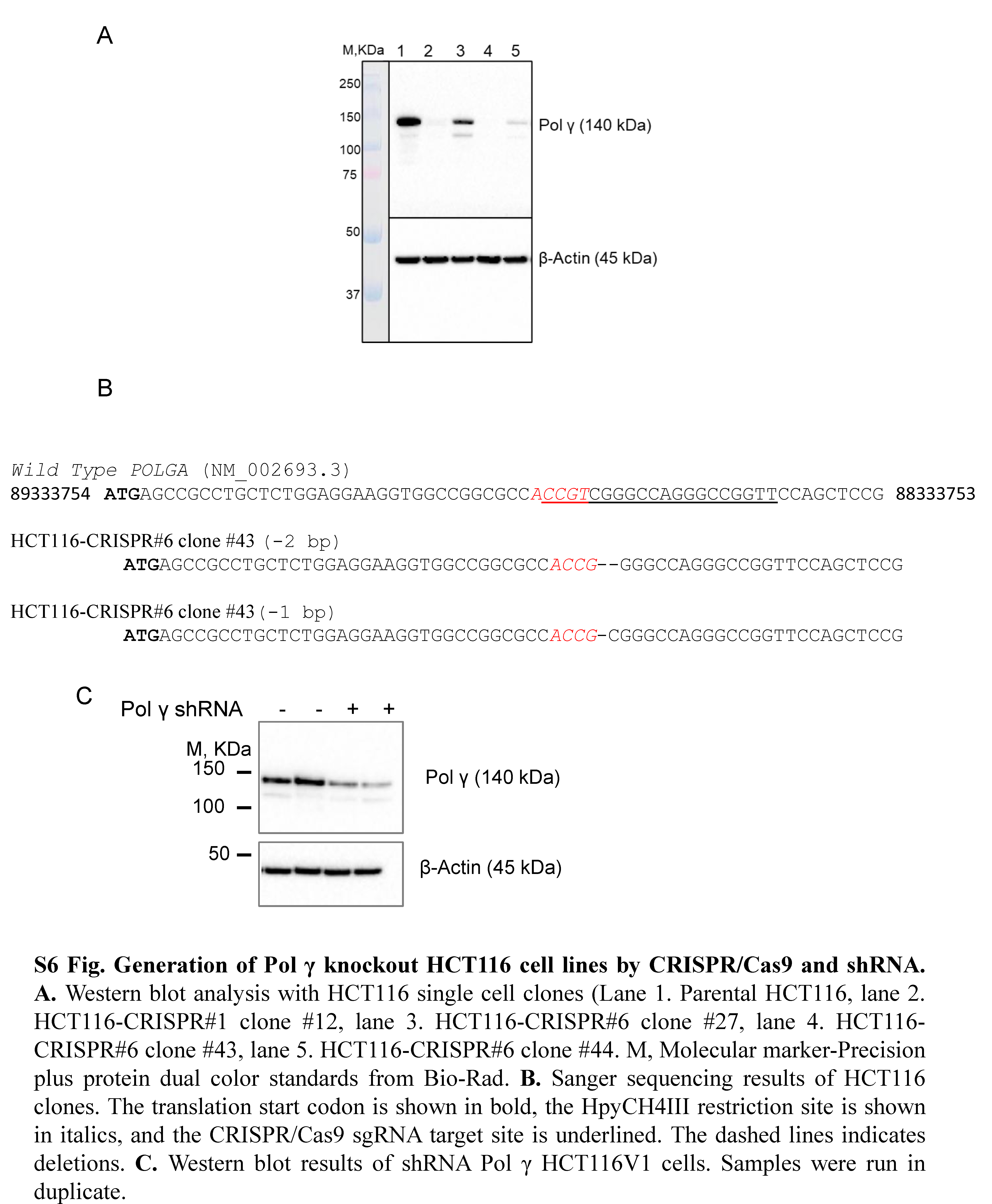

Supplement: S6 Fig — A. Western blot analysis with HCT116 single cell clones (Lane 1. Parental HCT116, lane 2. HCT116-CRISPR#1 clone #12, lane 3. HCT116-CRISPR#6 clone #27, lane 4. HCT116-CRISPR#6 clone #43, lane 5. HCT116-CRISPR#6 clone #44. M, Molecular marker-Precision plus protein dual color standards from Bio-Rad. B. Sanger sequencing results of HCT116 clones. The translation start codon is shown in bold, the HpyCH4III restriction site is shown in italics, and the CRISPR/Cas9 sgRNA target site is underlined. The dashed lines indicates deletions. (TIF) [file pone.0268391.s008.tif]

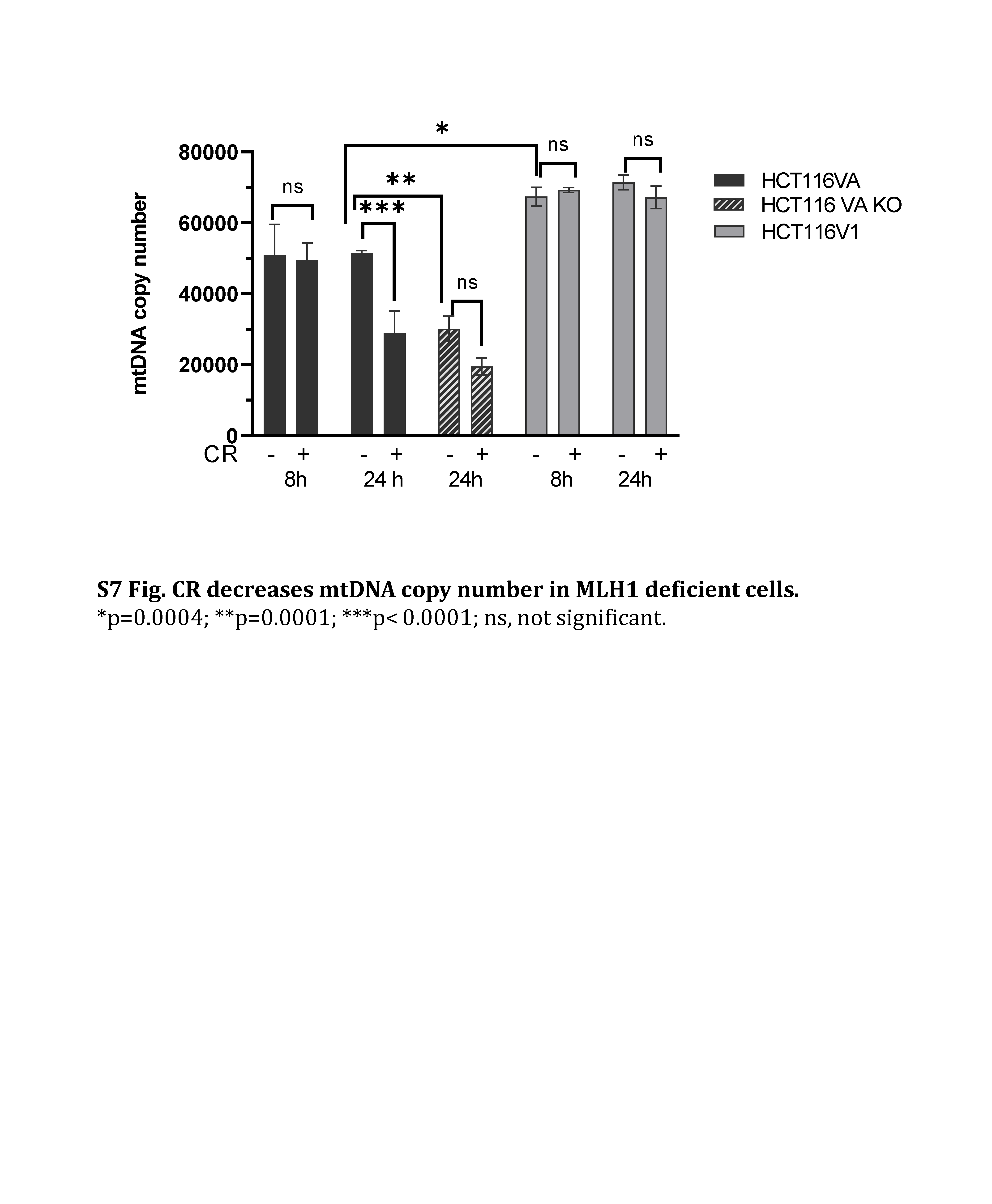

Supplement: S7 Fig — *p = 0.0004; **p = 0.0001; ***p< 0.0001; ns, not significant. (TIF) [file pone.0268391.s009.tif]
